# Comprehensive Two-Dimensional Gas Chromatography as a Powerful Strategy for the Exploration of *Broas* Volatile Composition

**DOI:** 10.3390/molecules27092728

**Published:** 2022-04-23

**Authors:** Andreia Bento-Silva, Noélia Duarte, Magda Santos, Carina Pedrosa Costa, Maria Carlota Vaz Patto, Sílvia M. Rocha, Maria Rosário Bronze

**Affiliations:** 1 FCT NOVA, Faculdade de Ciências e Tecnologia, Universidade Nova de Lisboa, Campus da Caparica, 2829-516 Caparica, Portugal; abentosilva@ff.ulisboa.pt; 2 iMed.ULisboa, Instituto de Investigação do Medicamento, Faculdade de Farmácia, Universidade de Lisboa, Avenida Prof. Gama Pinto, 1649-003 Lisboa, Portugal; mduarte@ff.ulisboa.pt; 3 DCFM, Departamento de Ciências Farmacêuticas e do Medicamento, Faculdade de Farmácia da Universidade de Lisboa, Av. das Forças Armadas, 1649-003 Lisboa, Portugal; 4 ITQB NOVA, Instituto de Tecnologia Química e Biológica António Xavier, Universidade Nova de Lisboa, Avenida da República, 2780-157 Oeiras, Portugal; cpatto@itqb.unl.pt; 5 Department of Chemistry & LAQV-REQUIMTE, Universidade de Aveiro, Campus Universitário Santiago, 3810-193 Aveiro, Portugal; magdasantos@ua.pt (M.S.); carina.pedrosa@ua.pt (C.P.C.); smrocha@ua.pt (S.M.R.); 6 iBET, Instituto de Biologia Experimental e Tecnológica, Avenida da República, Quinta do Marquês, Estação Agronómica Nacional, Apartado 12, 2780-157 Oeiras, Portugal

**Keywords:** maize, *broa*, maize bread, volatiles, Maillard reaction, baking, comprehensive two-dimensional gas chromatography

## Abstract

*Broa* is a Portuguese maize bread with characteristic sensory attributes that can only be achieved using traditional maize varieties. This study intends to disclose the volatile compounds that are mainly associated with the baking process of *broas,* which can be important contributors to their aroma. Twelve *broas* were prepared from twelve maize flours (eleven traditional maize varieties and one commercial hybrid). Their volatile compounds were analyzed by GC×GC–ToFMS (two-dimensional gas chromatography coupled with time-of-flight mass spectrometry) for an untargeted screening of the chemical compounds mainly formed during baking. It was possible to identify 128 volatiles that belonged to the main chemical families formed during this stage. Among these, only 16 had been previously detected in *broas*. The most abundant were furans, furanones, and pyranones, but the most relevant for the aroma of *broas* were ascribed to sulfur-containing compounds, in particular dimethyl trisulfide and methanethiol. Pyrazines might contribute negatively to the aroma of *broas* since they were present in higher amounts in the commercial *broa*. This work constitutes the most detailed study of the characterization of *broas* volatile compounds, particularly those formed during the Maillard reaction. These findings may contribute to the characterization of other maize-based foodstuffs, ultimately improving the production of foods with better sensory features.

## 1. Introduction

Bread is considered one of the most important foodstuffs worldwide [1]. In Portugal, whole maize flour is used to produce a traditional bread known as *broa* [2], considered one of the 50 world’s best breads by CNN Travel in 2019 [3]. Over the last centuries, several traditional open-pollinated maize varieties have been developed to produce high-quality *broas* with improved flavors and aromas that are not possible to achieve using the presently available more productive commercial maize hybrids [2,4]. 

The aroma of bread plays a key role in its acceptance by consumers [1,5,6]. The nature of aroma is very complex [7,8], and the contribution of an individual compound to the overall aroma depends on several factors, such as its odor description and threshold (the intensity perceived by olfaction), adsorption to the food matrices, content, and interactions with other volatiles [5,7,9,10,11]. In addition, the sensory description of a volatile may change depending on its concentration, therefore influencing, positively or negatively, the overall food aroma [12]. Only a small portion of the volatile compounds contribute to the overall aroma of bread [5,7,8,10,11,12]. These volatiles are often referred to as “key” or “character-impact” compounds [11,13,14] and are generally present at trace levels [5,15], while other compounds present in high concentrations are only scarcely perceived by the human nose [5]. Frequently, many potent aroma compounds must blend to give the integrated aroma and flavor perception of a certain food [16].

**Figure 1:**
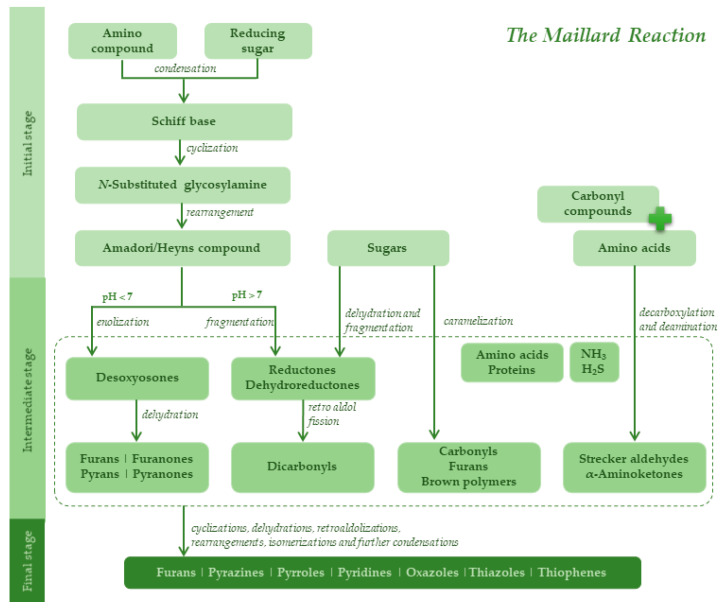
Representative scheme of the Maillard reaction [7,10,15,20,21,24,25,26].

The origin of bread volatiles is difficult to determine [17] once they result from lipid oxidation, fermentation and baking reactions [1,11]. However, at the baking stage, some of the most valuable aroma impact compounds are generated since they generally have low odor thresholds [18] and desirable sensory characteristics [7,9,15,19,20,21]. These reactions are often quoted as nonenzymatic browning reactions [21] and include mainly the Maillard reaction and, to a lower extent, the caramelization reactions [7,11]. Around 2 to 3% of sugars present in the dough undergo caramelization [22], giving rise to carbonyl compounds, furans, and brown-colored complex polymers [5,7,11,14,21,22]. The more relevant Maillard reaction occurs between carbonyls (most often reducing sugars) and free amino groups of amino acids, peptides, or proteins [5,7,9,18,20,23]. The main steps of this reaction are summarized in Figure 1. In the initial stage, free amino groups and reducing sugars condense and originate the Amadori or Heyns products [20,24]. Then, at the intermediate stage, furans, furanones, pyrans and pyranones are formed [20,24], following by amino acid degradation (Strecker reaction), when volatile compounds are formed without the need for sugars [25]. Ultimately, condensation and polymerization occur, leading to highly colored melanoidins and several aroma compounds, including pyrazines, pyrroles, pyridines, furans, oxazoles, thiazoles and thiophenes [7,11,15,20,21,26]. A previous study on *broas* has suggested that the volatiles produced at the baking stage, special pyranones, may positively contribute to the taste and aroma of this ethnical bread [27]. 

The volatile composition of bread has been usually characterized by HS-SPMS-GC-MS techniques (1D-GC) [27]. However, taking into account the complexity of bread aroma and the presence of very low amounts of important key volatiles [5,7,8,10,11], an analysis with a highly sensitive technique, such as comprehensive two-dimensional gas chromatography (GC×GC) [13,28], may allow the detection of important Maillard volatiles, which would be otherwise difficult or even impossible to identify. GC×GC employs two independent columns to separate sample analytes, and, therefore, the separation potential is greatly enhanced. ToFMS (time-of-flight mass spectrometry) detects unambiguous identification and ensures high selectivity throughout the chromatogram [28]. GC×GC–ToFMS has been recently used for untargeted food analyses, such as fruits, rice, hazelnuts, coffee and beverages [29,30]. To the best of our knowledge, this technique was only employed for targeted analysis of 2-acetyl-1-pyrroline in wheat and gluten-free bread [31] and has never been used to characterize the volatile composition of bread comprehensively. 

Taking advantage of the powerful GC×GC–ToFMS technique, the present research involved an in-depth investigation of the volatile compounds of *broas*, aiming at the disclosure of those that might be important contributors to the typical aroma of this ethnic bread. The knowledge of *broas* volatiles may also be useful for identifying relevant compounds in other foods, which currently remain unidentified. Ultimately, the data obtained in this work can be explored to fingerprint [29] *broas* prepared from traditional varieties and contribute to a better knowledge of the Maillard reaction, which is essential to design foods that present sensory attributes demanded by the consumers [15,31].

## 2. Results and Discussion

**Figure 2:**
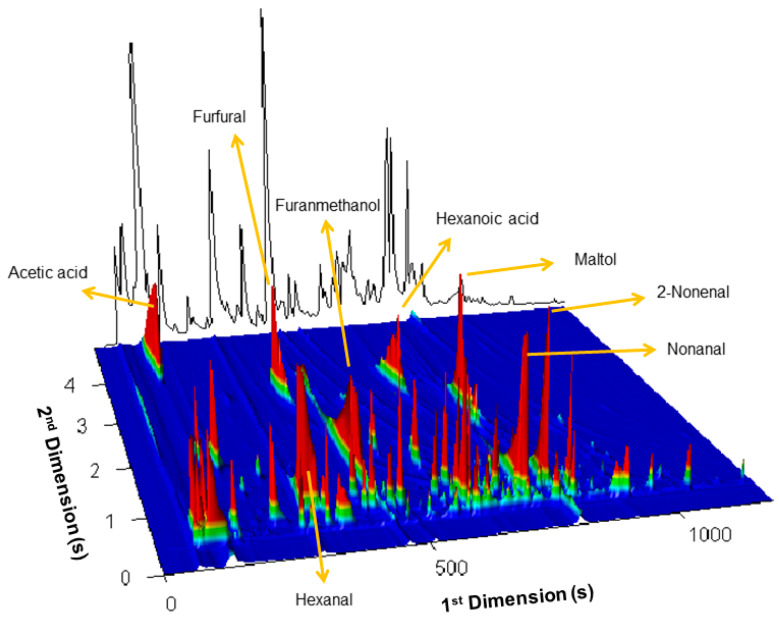
Contour plot of the total ion current GC×GC chromatogram of a broa sample (B1) under study. The highlighted volatiles have been previously detected in broas by GC-MS (1D) [27].

Eleven *broas* (B1 to B11) prepared from eleven traditional maize varieties and one *broa* (B12) prepared from commercial hybrid maize flour were studied (Table S1 and Figure S2) [27]. A contour plot of the total ion current chromatogram of a *broa* sample (B1) under study is presented in Figure 2. In a first approach, the presence of volatiles belonging to the chemical families mostly associated with baking was identified in the chromatograms of all studied samples (*n* = 12). A total number of 128 volatile compounds belonging to these families were detected and are described in Table 1. More than 80% of the compounds have been identified according to the criteria described in Section 3.3. 

The peak areas corresponding to the studied volatiles were measured, and their average in the twelve studied *broas* is presented in Table 1. The ratio of the peak area to odor threshold values in water (OT, in the Log_10_ form) was used as a screening method to identify the most relevant volatiles to the aroma of *broas*. Calculation of the odor activity value (OAV, ratio of the concentration to OT) is often carried out to evaluate the most aroma-active compounds in a food product. A compound might be sensed when OAV > 1 (or >0, when the Log_10_ form is considered) [18,30,32,33]. Although differences in peak areas among different compounds do not give direct information about their relative concentration, they greatly varied (sometimes more than 10.000-fold), suggesting they were present in very distinct concentrations. Thus, the higher the ratio, the higher the probability of a compound contributing to the aroma of this bread. The table also includes some examples of foods where the compounds have been detected (visually represented in Figure S1 from Supplementary Materials).

The results will be presented as follows: firstly, the advantages of the analysis of *broas* volatile compounds by GC×GC–ToFMS will be discussed by providing examples that allow the identification of unreported compounds. Secondly, the compounds will be explored according to their chemical families, and those which may be more relevant to the aroma of *broas* will be suggested. Finally, the results from the analyzed samples will be compared, and the correlations among their volatiles will be studied to elucidate which compounds may be responsible for the differences in their aroma.

### 2.1. The potentialities of GC×GC–ToFMS in the Identification of Broas Volatile Compounds

**Figure 3:**
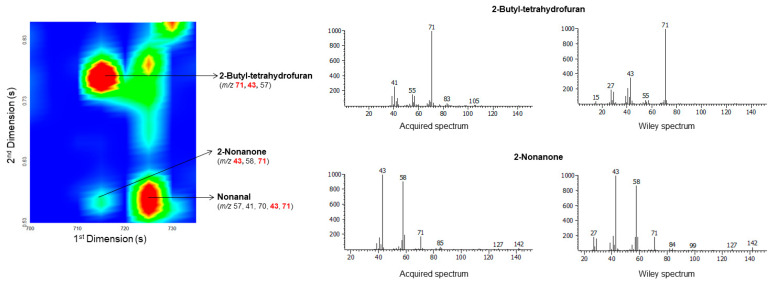
A blow-up of a part of a contour plot extracted ion chromatogram at *m/z* 71 from a *broa* sample, showing the separation, through the ^2^D column, of 2-nonanone and 2-butyl-tetrahydrofuran, which showed the same retention time in the ^1^D column and shared similar mass spectra.

In a previous study on *broas* volatile composition by GC-MS (1D) [27], only 16 out of the 128 volatiles described in the present work were detected (Table 1), which confirms that GC×GC–ToFMS provides a much higher potential for the detection and identification of food volatiles [63,64]. It should also be noted that, in this work, a relatively high signal-to-noise (S/N) threshold (100) was used, which limited the number of detected compounds. Figure 2 and Figure 3 illustrate the advantages of GC×GC–ToFMS, which allowed the separation of analytes with similar volatility and common product ions through the secondary ^2^D column. Considering the set of columns used (non-polar/polar), the decrease in volatility (high ^1^t_R_) is mainly related to the increase in the number of carbons [64,65]. In contrast, the increase in the ^2^t_R_ corresponds to increasing polarity [64,65]. For instance, 2-butyl-tetrahydrofuran (F31) was not detected in the previous study [27]. This compound was co-eluted with 2-nonanone (^1^t_R_: 715 s), and both presented similar mass spectra, with identical product ions at *m/z* 71 and 43 (Figure 3). However, since they present different polarities, their separation was achieved through the ^2^D column. 2-Butyl-tetrahydrofuran (F31) exhibits a slightly lower Log P than 2-nonanone (Log P: 2.93 and 3.08, respectively), which supports its relatively high polarity, and, therefore, the higher retention time in the ^2^D (^2^t_R:_ 0.77 vs. 0.56 s). Thus, despite the high similarity index of 2-nonanone with the library spectra (89%) obtained in the previous analysis by GC-MS [27], the compound 2-butyl-tetrahydrofuran also contributed to the peak area considered for 2-nonanone. Without the high resolving power of GC×GC, the separation, identification, and relative quantitation of these two compounds would have been a challenge.

**Figure 4:**
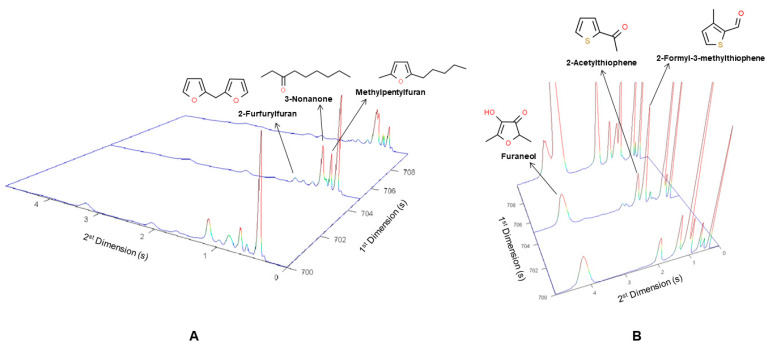
A blow-up of (**A**) total ion GC×GC chromatogram and (**B**) extracted ion chromatogram at *m/z* 126 and 128, through the ^2^D column from a *broa* sample, showing the separation of methylpentylfuran (F27), furfurylfuran (F28), 2-formyl-3-methylthiophene (Tp4), 2-acetylthiophene (Tp5) and furaneol (Fo20).

Another example is the identification of methylpentylfuran (F27). This compound was not commonly detected in foods but was previously tentatively identified in *broas* by GC-MS [27]. The results obtained in the present work corroborate this earlier identification. The mass spectrum of F27 showed a high similarity index with the spectra from methylpentylfuran from the Wiley database (Table 1). Furthermore, this compound showed a very low retention time of 0.50 s in the ^2^D column (Figure 4), suggesting its low polarity, which was confirmed by its calculated Log P of 4.43. Although methylpentylfuran usually refers to 3-methyl-2-pentylfuran, F27 can also correspond to other isomers, such as 2-methyl-5-pentylfuran or 4-methyl-2-pentylfuran. In addition, several other volatiles belonging to chemical families, which usually arise during baking, were eluted at this ^1^t_R_ (705 s), namely furfurylfuran (F28), 2-formyl-3-methylthiophene (Tp4), 2-acetylthiophene (Tp5) and furaneol (Fo20). Since they show different polarities (Log P of 2.52, 1.38, 1.28 and –0.33, respectively), they were separated through the ^2^D column (^2^t_R_: 0.95, 1.31, 1.56 and 4.02, respectively). As shown in Figure 4A, the peak intensities of methylpentylfuran (F27) and 3-nonanone were much higher than F28, Tp4, Tp5 and Fo20. Thus, it would not have been possible to identify these compounds without the orthogonal separation through the ^2^D column [64,65]. The identification of volatile sulfur compounds (Figure 4B) is particularly relevant since they are typically present in foods at extremely low levels, often at sub-parts-per-billion concentrations, but provide background sensory nuances to the flavor and are often considered “character-impact compounds” [13].

These results show the potentialities of GC×GC–ToFMS for the characterization of food volatiles and demonstrate the complexity of the volatile composition of *broas*. Moreover, this technique also allowed the identification of several compounds belonging to the characteristic chemical classes of the baking process, which have not been commonly described either in bread or maize-based foods. 

### 2.2. Exploring the Volatile Compounds Associated with Baking

The most relevant compounds of *broas*, selected based on their abundance and, more importantly, their OTs, will be discussed below.

#### 2.2.1. Furans and Furanones

Furan and furanone derivatives are among the most common volatiles of heated foods [20,66], and give burnt, savory, sweet and caramel aroma to foods [9,15,20]. The major furans included 2-pentylfuran (F20) and 2-butylfuran (F14), which have been previously detected in *broas* [27]. 2-Pentylfuran (F20) was particularly abundant, and it is a potent odorant [48], giving floral-fruity notes [60], which may significantly contribute to their aroma. It has been reported as the most common aroma-active furan in wheat bread crumb [60] and a likely contributor to the total aroma and flavor of maize tortilla chips [43] and popcorn [33]. 

Several furans with oxygenated substituents, such as furfurals and furanones, were also identified in *broas*. Among all the compounds described in the present work, the highest peak areas were obtained for 2-furanmethanol (F13). Although it is not a very strong odorant [32,48], this compound may also be relevant for the aroma of *broas* due to its abundance. It is usually associated with pleasant, creamy and caramel notes [41] and may be relevant to the aroma of popcorn [33]. Other oxygenated furans include 5-methyl-2-furanmethanol (F19), 3-furfural (F10), and isomaltol (F22), which had not been previously reported in *broas* [27]. Among these, 5-methyl-2-furanmethanol showed a relatively low OT and was confirmed as a key aroma compound of Chinese white bread [49]. It was not possible to confirm the OTs of 3-furfural and isomaltol, but they have been considered important compounds to the aroma of popcorn [33] and wheat bread [5,21], respectively. 

Only two out of the twenty-six furanones detected in the present work have been previously detected in *broas*, namely *N*-caprolactone (Fo19) and γ-nonalactone (Fo26). Both are possible contributors to *broa’s* sweet aroma [5,41] since they were present in high amounts in *broas* and are potent odorants [44,47]. γ-Octalactone (Fo23), detected in *broas* for the first time, may also contribute to their aroma, considering its abundance and low OT.

#### 2.2.2. Pyrans and Pyranones

Pyrans have not been described in similar food products, but some may also be important odorants, specially tetrahydropyrans [48]. In contrast, pyranones occur in the volatiles of all heated foods [20], conferring sweet, burnt, pungent and caramel-like flavors and aromas [15]. The most relevant pyranones were maltol (Po7) and 3-hydroxy-2,3-dihydromaltol (Po8). These compounds have been previously described as possible positive contributors to the ‘taste and aroma’ of *broas* [27]. Maltol imparts desirable, caramel-like, sweet and fruity characteristics to foods [14,20,21]. It can mask bitter flavors [14] and is considered a key odorant in cereal products [67]. Although it has a relatively high OT of 2500–9000 µg L^−1^ [14,43], it may be important for the aroma of *broas* due to its abundance. 

#### 2.2.3. Pyrazines

In the present study, twelve pyrazines were identified in *broas*, although only 2-methyl pyrazine (Pz2) had been previously described [27]. Pyrazines are considered impact odorants, with characteristic pleasant nutty and roasted odor notes [7,11,20,59,68]. They significantly contribute to the flavor of baked products [11], such as bread [5,7,31], popcorn, rye crisp bread [68] and maize products [24]. *Broas* appear to have a lack of pyrazines when compared to similar foods. Recent studies have shown that the amount of pyrazines varies greatly between different cereal bread [31,56,69]. 

Although the OT values of alkylpyrazines are relatively high (above 1000 µg L^−1^) [20,68], replacing one or more methyl groups with ethyl can give a marked decrease in the OT. Some ethyl-substituted pyrazines have sufficiently low threshold values to be important in the roast aroma of cooked foods [20]. Taking into account the OTs described for pyrazines and their amount in *broas*, 2-methylpyrazine (Pz2), 2,6-dimethylpyrazine (Pz3) and, especially, 2-ethyl-dimethylpyrazine (Pz9), may be relevant for their overall aroma. The former shows nutty, cocoa, and roasted meat aromas [68]; however, it was described as a possible off-volatile popcorn [33]. 2,6-Dimethylpyrazine was also considered an important volatile in maize meal extruded product with whey protein [24]. Lastly, Pz9 was tentatively identified as 2-ethyl-3,6-dimethylpyrazine or 2-ethyl-3,5-dimethyl-pyrazine since they show similar SI and LRI (Table 1). Both compounds are potent odorants and contribute to cocoa, nutty, potato and roasted notes [51]. 2-Ethyl-3,5-dimethylpyrazine is a key odorant of maize tortilla chips, popcorn and rye bread crust [7,43,70], whilst 2-ethyl-3,6-dimethylpyrazine is a key odorant of maize tortilla chips, taco shell [32,43] and popcorn [33]. 

Some pyrazines, such as 2-acetyl-3-methylpyrazine (Pz10), 5*H*-5-methyl-6,7-dihydrocyclopentapyrazine (Pz11) and 2-(2′-furyl)-pyrazine (Pz12), have not been detected in related food products, but may still be relevant to the aroma of *broas*, since Pz10 and Pz11 may give maize-like aromas to foods [51]. 

#### 2.2.4. Pyridines and Pyrimidines

Pyridines and pyrimidines were detected in *broas* for the first time. Although they were not present in high amounts, they might still be relevant to the overall aroma due to their low OTs. Pyridines give roasted and popcorn odors to foods [14,43]. 

2-Acetyltetrahydropyridines (or 6-acetyltetrahydropyridines) are possibly the most common pyridines in similar foodstuffs. They are potent Maillard flavor compounds that contribute substantially to caramel, roasty, and bready aromas [51,71] in several bakery products [14,16,32,71], and are formed by the degradation of proline and hydroxyproline [20]. However, these compounds were not detected in *broas*. Instead, 1-acetyl-1,2,3,4-tetrahydropyridine (Pd8) was identified as the main pyridine. Although it exhibits a similar mass spectrum to 2-acetyltetrahydropyridine, Pd8 showed a very intense peak at *m/z* 68 (Figure S3), which is not expected in 2-acetyltetrahydropyridine [72]. This compound has also been identified in the crusts of wheat bread [42] and lupin protein isolate-enriched wheat bread as an important contributor to its aroma profile [42]. Thus, it might also be an important *broa* volatile, contributing to a nutty odor [42].

2-Pentylpyridine (Pd9) may also be relevant for *broas* aroma, contributing to roasted and nutty odors [51]. Although it was present in lower amounts than other pyridines, it shows a very low OT [48]. This compound has not been described in similar foods, probably due to its low concentration, but it has been described in several fried foods [73]. 

4-Methylpyrimidine (Pd2) was the only pyrimidine detected in *broas*. This compound has not been described either in bread or in other maize-based foods, and it was not possible to obtain any information regarding its odor characteristics.

#### 2.2.5. Pyrroles, Pyrrolines and Oxazoles

Pyrrole derivatives are responsible for roasted odors [14]. Skatole (Py12) has a very low OT of 0.2 µg L^−1^ [16] and may be relevant to the aroma of *broas*. It is usually described as an off-flavor. However, in low concentrations, it may introduce a natural note of ‘overmature flower’ [51].

One of the most striking results to emerge from this study was the absence of pyrrolines in *broas*. These compounds are abundant bread volatiles, significantly contributing to their flavor [5,7]. Pyrrolines have been described as character impact odorants of the ‘roasted’ and ‘popcorn-like’ notes [7]. Important pyrrolidines described in cereal bread and maize-based foods are 2-acetyl-1-pyrroline and its precursor 1-pyrroline [7,14,20,59]. However, recent studies have shown that 2-acetyl-1-pyrroline varies greatly among different cereal bread [69]. For instance, 2-acetyl-1-pyrroline seems not to be so relevant to the aroma properties of rye bread [7], and it was not detected in gluten-free bread, which was analyzed by GC×GC [31]. By contrast, other authors have found that 2-acetyl-1-pyrroline was higher in gluten-free bread [56]. These differences can be explained by the presence or absence of precursors or interferents in the cereal flours. For instance, differences in the ornithine content of yeasts may play a role in forming pyrrolines since this amino acid has been ascribed as the most important precursor for forming 2-acetyl-1-pyrroline during baking [7,11,20]. 2-Acetylpyrroline may also be formed by the degradation of proline and hydroxyproline, similar to 2-acetyl-tetrahydropyridines [20], which were also not found in *broas*, as previously discussed. Thus, differences in the amounts of proline and hydroxyproline may also influence the production of 2-acetylpyrroline and 2-acetyl-tetrahydropyridines. In addition, phenolic acids may inhibit the production of 2-acetyl-1-pyrroline [74], and whole maize flours were used to prepare broas, which have higher amounts of phenolic compounds compared to other cereals or with refined maize flours [75]. Lastly, 2-acetyl-1-pyrroline is highly volatile and can be oxidized to 2-acetylpyrrole [14,20], one of the major volatile compounds detected in the present work.

Three oxazoles were detected in *broas*, but they probably have a low impact on their overall aroma, as reported for other foods [20]. They were present in *broas* in very low amounts compared to other compounds.

#### 2.2.6. Sulfur-Containing Compounds

Sulfur compounds had not been previously reported in broas [27], possibly due to their low concentrations. Usually, foods from cereals show very low contents in sulfur-containing compounds due to the low amounts of sulfur amino acids [20]. However, sulfur-containing Maillard odorants constitute the most powerful aroma compounds of foods, even though they are present at trace levels [14,15]. These compounds have traditionally been associated with unpleasant and noxious off-flavors [13,14]. Still, they contribute to positive flavor characteristics at low concentrations (<1 µg kg^−1^), giving tropical, fruity, and savory aromas to foods [13]. They are considered “character-impact compounds” of bread crust, popcorn and toasted cereal grains [13,15].

Dimethyl sulfide (S2), dimethyl disulfide (S3), and, in particular, dimethyl trisulfide (S8) may be extremely relevant for the aroma of *broas*. At low levels, they all show positive aroma characteristics. Dimethyl trisulfide (S8) has an extremely low OT of 0.01 µg L^−1^ [43] and, when present at low concentrations, is associated with tropical fruit and grapefruit aromas [13]. The most abundant sulfur compound detected in *broas* was dimethyl disulfide (S3), an important contributor to maize flavor [24]. Dimethyl sulfide (S2) is considered a flavor impact compound of sweet maize and conveys the typical flavor impression of canned maize when present at reduced levels [13]. Methanethiol (S1) was the second most abundant sulfur-containing compound and may also significantly impact the aroma of *broas*. It has been described as a possible contributor to the aroma of sweet maize [13].

Another sulfur-containing compound detected in *broas* was the very well-known methional (S7) [68], possibly also one of the most relevant volatiles for the aroma of *broas*. It is a potent [5] and desired odorant [21], with a characteristic potato-like aroma [7,59]. It has been considered a key odorant of fried foods [43], wheat, rye [7] and Chinese white [49] bread. It may be responsible in part for typical popcorn [33], maize tortilla chips [43] and taco shell [32] aroma characteristics. Furthermore, it has flavor-modifying characteristics since it has been shown to suppress flavor and aroma [59]. Other aliphatic sulfur compounds detected in *broas* were 1-methylthiopropane (S4) and methylthio-2-propanone (S5). These compounds have not been described in similar foods, and their impact on the overall aroma of *broas* is unknown. 

Regarding aromatic sulfur heterocycles derivatives, it was possible to detect three thiazoles and six thiophenes. Thiazoles usually confer green, vegetable-like, cocoa and nutty aromas to foods [20], while thiophenes contribute to sulfurous, nutty and fatty aromas [51]. Among them, 2-acetylthiazole (Tz2) may have high importance in the aroma of *broas*. It has been described as a key volatile of popcorn and maize flour extrudates [40]. It was possible to detect six thiophenes in *broas*. Still, they have not been so commonly described in similar foods, and it was not possible to find information regarding their OTs in the literature. 

Another sulfur heterocycle compound detected in *broas* was an oxathiane derivative (S6), which may refer to 1,2-, 1,3- or 1,4-oxathiane. However, considering their polarities (Log P of 1.14, 0.43 and 0.36, respectively) and the ^2^t_R_ of S6 (1.10 s), it was tentatively identified as 1,2-oxathiane. This compound has not been described in foods, but oxathiane derivatives have been ascribed as important aroma compounds. The most relevant example is 2-methyl-4-propyl-1,3-oxathiane, a key aroma compound of passion fruit, with a low OT of 3 µg L^−1^ [13]. Recently, this compound and 2,4,4,6-tetramethyl-1,3-oxathiane have been detected in wines [76]. Although the impact of oxathiane on the aroma of foods is currently unknown, it may impact the aroma of *broas*, and this compound is worth further study. 

### 2.3. An Overall View of the Volatiles Associated with Baking in Broas

**Figure 5:**
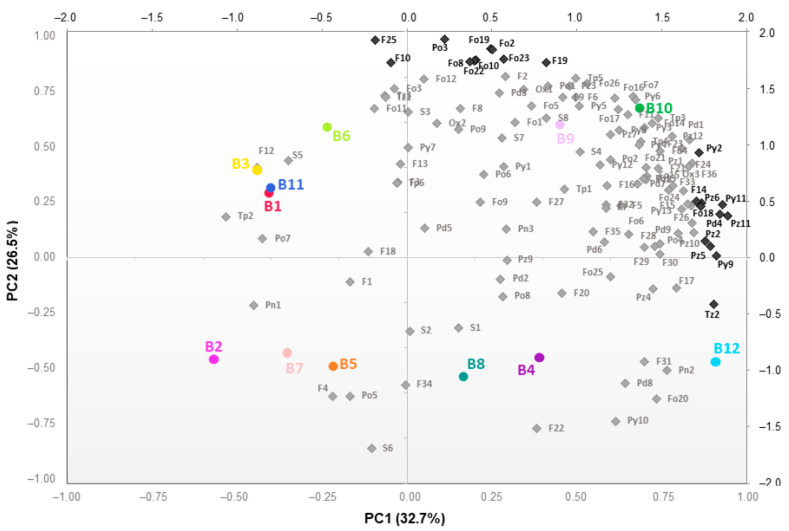
Projection of *broas* (coloured dots) and variables (grey) (Table 1) in the plane defined by PC1 and PC2, corresponding to 59.2% of the total variance. The volatiles that strongly (PC > 0.85) contributed to differentiating the samples along the two PCs are represented in black.

Sensory analysis in a previous study [77] revealed that the *broa* prepared from the available commercial maize hybrid variety (B12) showed the lowest scores for ‘smell and odor’ and ‘taste and aroma’. In contrast, the traditional *broas* (obtained from the traditional open-pollinated varieties) were poorly discriminated among them [77]. A subsequent study revealed that these differences could at least be partially explained by their volatile composition [27]. Thus, a PCA (principal component analysis) was performed to explore further these differences for an easy, rapid and global assessment of the main differences in the volatile composition among the studied *broas*. Figure 5 shows the projection of samples and variables in the space defined by the two principal components, corresponding to 59.2% of the total variance. A hierarchical cluster analysis combined with a heatmap representation was also constructed and shown in Figure S4. 

**Figure 6:**
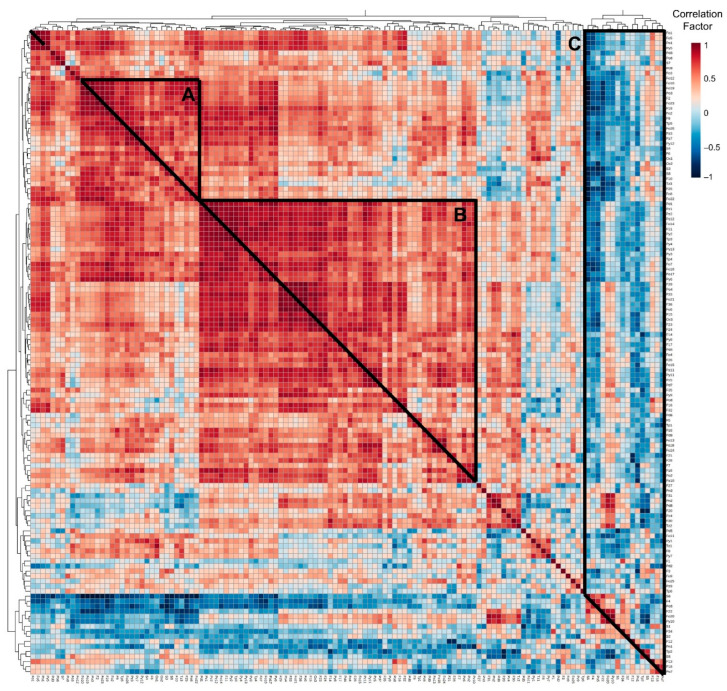
Correlation heatmap of the 128 volatile compounds in *broas*. The correlation coefficients of volatiles are represented through a chromatic scale from deep blue (−1), corresponding to negative correlations, to red (1), corresponding to positive correlations. The letters correspond to volatiles that (**A**) strongly contributed to differentiating the samples along PC2; (**B**) strongly contributed to differentiating the samples along PC1 and (**C**) negatively contributed to PC1 and PC2.

Since all *broas* were prepared following the same procedure and submitted to the same temperature of kneading and baking, it can be stated that the differences observed among the different *broas* were caused by differences in the corresponding maize flours, which affect the Maillard reaction. A matrix correlation analysis was conducted and represented by a heatmap to shed some light on the volatile compounds which might have been formed from the same precursors (Figure 6 and Tables S4 and S5). 

The volatiles that strongly contributed to differentiating the samples along PC2 (Figure 5) are represented in group A of the correlation matrix (Figure 6) and highlighted in Tables S4 and S5. This group is characterized by a simple pattern of strong and positive correlations amongst several furans and furanones. In low-moisture starchy foods, such as *broas*, the Maillard reaction seems to be the main route of their formation [78,79]. Thus, higher amounts of the Maillard reaction precursors, such as amino acids and sugars, may have generated *broas* with higher amounts in furans. Alternatively, the presence of interferents, for instance, phenolic compounds [27], may explain these correlations since they may promote [27,80] or inhibit [74,80] the reaction. In particular, very strong correlations (R > 0.85, *p* < 0.05) were observed among 2-methylfuran (F2), 5-methyl-2-furanmethanol (F19) and 2-acetyl-5-methylfuran (F25), which are usually produced by caramelization reactions and by the breakdown of the Amadori or Heyns intermediates in the early stages of the Maillard reaction (Figure 1) [14,19,20]. Strong and positive (R > 0.7, *p* < 0.05) correlations were also found between dihydro-2-methyl-3-(2*H*)-furanone (Fo2) and sulfur-containing heterocycles. Higher amounts in dihydro-2-methyl-3-(2*H*)-furanone, which reacts with ammonia and hydrogen sulfide (produced from cysteine by hydrolysis or by Strecker degradation) [19,20,24,81], may directly cause higher amounts of thiazoles and thiophenes.

**Figure 7:**
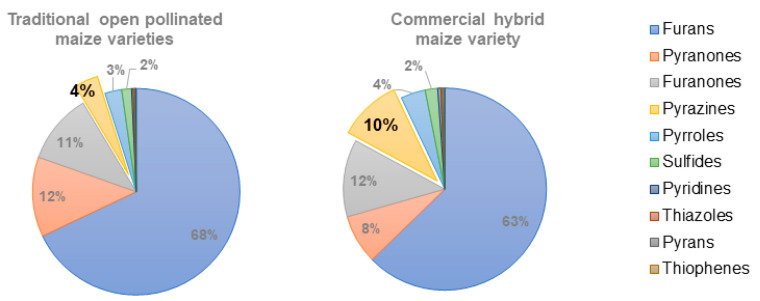
Representation of the percentage of chromatogram area for the families of chemical compounds studied in *broas* prepared from traditional maize varieties (*n* = 11) and commercial maize flour.

The volatile compounds that strongly contributed to differentiating the samples along PC1 (Figure 5) are placed in group B of the correlation heatmap (Figure 6 and Tables S4 and S5) and consisted, among others, of several pyrazines. The major route for the formation of pyrazines is thought to be from α-aminoketones, which are products of the condensation of a dicarbonyl with an amino compound via Strecker degradation during the Maillard reaction [20,24]. Very strong and positive correlations were found between Pz7 (2-methyl-5-vinylpyrazine) and Pz3 (2,6-dimethylpyrazine), both products from the reaction between leucine and fructose [59]. The *broa* prepared from the available commercial hybrid variety maize flour (B12) showed a higher content in pyrazines (*p* < 0.05) than all the traditional varieties (Table S2 and Figure 7), especially in 2-methylpyrazine (Pz2) and 3-dimethylpyrazine (Pz5) (Table S3). The higher amounts of total ferulic acid in the traditional maize varieties [82] might have inhibited the formation of pyrazines [80] in the corresponding *broas*. 

Lastly, the volatiles which negatively contributed to PC1 and PC2 are located in group C (Figure 6). These volatiles show strong and negative correlations with several of the volatiles which belong to groups A and B. Furfural (F12) was strongly and negatively correlated to both benzofuran (F21) and furfurylfuran (F28). Thus, *broas* with higher amounts of furfural also showed lower amounts of benzofuran and furfurylfuran, and vice versa. Furfural is mainly generated in the initial stages of the Maillard reaction and participates in further reactions [14,24,83]; thus, differences in the precursors which reacted with furfural and generated benzofuran and furfurylfuran, or the presence of interferents of this reaction [27,80], may have influenced its extent. Similarly, negative correlations were found among several sulfur compounds and both furans and furanones, which have been described as important precursors of thiazoles and thiophenes [24], and between oxathiane (S6) and sulfur-containing compounds, such as dimethyl disulfide (S3), dimethyl trisulfide (S8), 2-acetylthiophene (Tp5) and benzothiazole (Tz3), which might have been precursors of the formation of oxathiane. A recent study in wines has proposed that thiols act as precursors of the formation of oxathiane [76].

Taken together, these results suggest that the main differences among the volatile compounds of the studied *broas* are likely due to the presence of specific precursors or interferents which affect the extent of some reaction pathways of the Maillard reaction, as the formation of pyrazines and oxathiane, rather than the overall Maillard reaction. These results can partially explain the contradictory reports on the effect of phenolic compounds on the Maillard reaction [27,74,80]. Differences in precursors or interferents of certain pathways of this reaction in maize varieties can cause differences in *broas* aroma.

As previously stated, the analyzed commercial hybrid variety sample (B12) showed lower scores in a sensory analysis [77]. The results from the present study have shown that this sample showed a higher content in pyrazines (Figure 7), particularly in 2-methylpyrazine (*p* < 0.05). These results were somewhat surprising since pyrazines are strong odorants usually associated with positive sensorial characteristics, giving roasted and nutty aromas to bread [5,51]. However, pyrazines may contribute to aroma characteristics in *broas* that are not typical of this type of bread, giving rise to some of the negative comments associated with B12, such as a ‘weak typical flavor’, ‘wheat bread flavor’, ‘with a weak maize flavor’ and ‘no history’ [27]. Similarly, it has been reported that most pyrazines contributed negatively to the aroma of popcorn [33]. Besides the higher amount in pyrazines, a closer examination of Table S3 also showed that 2-butyltetrahydrofuran (F31) was present in B12 in higher (*p* < 0.05) amounts. However, this compound has not been described in similar foods, and it was not possible to infer its impact on *broas* aroma. Therefore, these data should be further explored for the characterization of broas aroma and fingerprinting [29], since both 2-butyltetrahydrofuran and 2-methylpyrazine may be potential biomarkers of the authenticity of *broas* prepared from traditional maize varieties.

Other compounds can contribute to the lower sensory attributes of B12, namely the higher contents in some pyridines and pyrroles, including the powerful skatole (Py12), often associated with off-aromas [51], and lower contents in maltol (Po7), associated with higher ‘taste and aroma’ [27]. Although the ANOVA showed that these results were not statistically different from some traditional varieties (*p* > 0.05) (Table S3), combinations of volatiles can yield different characteristics than those expected from individual compounds [12,30], and therefore contribute to the lower sensory attributes of *broas* prepared from the commercial maize hybrid variety under study. 

## 3. Materials and Methods

### 3.1. Samples

Eleven *broas* (B1 to B11) were prepared from eleven traditional open-pollinated maize varieties, described in Table S1. The traditional samples were chosen as representative of the Portuguese maize germplasm variability, taking into account their agronomic performance in field trials, basic nutritional quality and genetic diversity evaluated under the scope of the FP7 SOLIBAM European project. One *broa* (B12) prepared from a commercial maize hybrid variety was also studied.

*Broas* was prepared following a traditional recipe, as previously described [27]. The ingredients included 70% maize flour, 20% commercial rye flour (Concordia type 70, Portugal) and 10% commercial wheat flour (National type 65, Portugal). The dough was manually molded into 400 g balls and baked in a gas oven (Matador; Werner & Pfleiderer) at 270 °C for 40 min.

### 3.2. HS-SPME Methodology

The HS-SPME methodology was based on the previous work developed by Bento-Silva et al., 2021 [27]. Briefly, 4 g of *broas* (whole bread, including crumb and crust) were smashed manually and placed in a 20 mL vial. A 50/30 µm DVB/CAR/PDMS (divinylbenzene/carboxen/polydimethylsiloxane) fiber purchased from Supelco (Sigma-Aldrich, St. Louis, MO, USA) was used to concentrate the volatile compounds present in the headspace. Samples were extracted for 40 min in a thermostated bath adjusted to 60.0 ± 0.1 °C at 250 rpm. All samples were extracted in duplicate. 

### 3.3. GC×GC–ToFMS Analysis

The GC×GC–ToFMS experimental parameters were adapted from Costa et al. (2020) [65]. The equipment used was a LECO Pegasus 4D GC×GC–ToFMS system (LECO, St. Joseph, MI, USA) consisting of an Agilent GC 7890A gas chromatograph (Agilent Technologies, Inc., Wilmington, DE, USA) with a dual-stage jet cryogenic modulator (licensed from Zoex) and a secondary oven, and a mass spectrometer equipped with a time-of-flight (ToF) analyzer.

The SPME fiber was manually introduced into the port at 250 °C for analyte desorption. The injection port was lined with a 0.75 mm I.D. glass liner. Splitless conditions (30 s) were used. An Equity-5 30 m × 0.32 mm I.D., 0.25 μm film thickness (Supelco, Bellefonte, PA, USA) was used as a first-dimension column (^1^D), and a DB-FFAP 0.79 m × 0.25 mm I.D., 0.25 μm film thickness (J&W Scientific Inc., Folsom, CA, USA) was used as a second-dimension column (^2^D). The carrier gas was helium at a constant flow rate of 2.50 mL min^−1^. The primary oven temperature was programmed from 35 °C (5 min) to 230 °C (2 min) at 10 °C min^−1^, and the secondary oven program was 30 °C offset above the primary one. The MS transfer line and MS source temperatures were set at 250 °C. The modulation period was 5 s, keeping the modulator at 20 °C offset above the primary oven, with hot and cold pulses by 0.80 and 1.70 s, respectively. The mass spectrometer ran in the EI mode at 70 eV and detector voltage of –1530 V, using an *m/z* range of 35–350.

In an initial approach, the total ion chromatograms were processed using the automated data processing software ChromaTOF (LECO) at the S/N threshold of 100. The obtained GC×GC total ion chromatogram contour plots exhibited more than 1200 peaks. As the present study focused on the compounds mainly produced during baking, in a second approach, all the compounds belonging to the characteristic chemical families associated with the Maillard and caramelization reactions were selected. In addition, the volatile compounds described in the literature as key aromas of bread or maize-based foods and which were not detected using the automated data processing (S/N < 100) were searched on the chromatograms based on extracted ion chromatogram contour plots of the characteristic ions, and the corresponding peak areas were also included. 

For identification purposes, the mass spectrum and retention times (^1^D and ^2^D) of the analytes were compared with the mass spectral libraries, namely an in-house library of standards and two commercial databases (Wiley 275 and US National Institute of Science and Technology (NIST) V. 2.0–Mainlib and Replib). Additionally, the linear retention index (LRI) was experimentally determined according to the van den Dool and Kratz LRI equation [84]. A C_8_-C_20_ *n*-alkane series was used for LRI determination (the solvent *n*-hexane was used as the C_6_ standard). These values were compared with those reported in the literature for chromatographic columns similar to the ^1^D column mentioned above. A positive identification was considered when the experimental spectra shared at least an 80% similarity with spectra from the software libraries and the LRI deviation was less than 5%. This difference in LRI takes into account that (i) the literature data are obtained from a large range of GC stationary phases (several commercial GC columns are composed of 5% phenylpolysilphenylene-siloxane or equivalent stationary phases), and (ii) the modulation causes some inaccuracy in the first dimension retention time, and the majority of the values reported in the literature were determined in a 1D-GC separation system [64]. The Log *p* values were calculated using ALOGPS 2.1 to confirm the identification of some compounds which did not match all the criteria described above [85].

The DTIC (deconvoluted total ion current) GC×GC peak area data were used to estimate the relative content of each volatile component in the different samples.

### 3.4. Statistical Analysis

The peak area data of all studied compounds were extracted from the chromatograms and used to build the full data matrix consisting of 12 *broas* samples and 128 variables. After data normalization, hierarchical cluster analysis (HCA) and Pearson’s coefficient correlations, both combined with heatmap visualizations, were applied for this dataset using the MetaboAnalyst 3.0 (web software, The Metabolomics Innovation Centre (TMIC), Edmonton, AB, Canada). Independent sample *t*-tests, ANOVA followed by post hoc Tukey tests and principal component analyses (PCA) were obtained using the software SPSS version 21 (IBM, Armonk, NY, USA). The limit of significance was set at *p* < 0.05.

## 4. Conclusions

This study aimed to extend previous research on the volatile compounds of broas, focusing on those mainly associated with the baking process. Almost 90% of the compounds identified in this work have not been previously detected in *broas*. One of the most relevant study findings was the absence of pyrrolidines and the lack of pyrazines in *broas*, especially when prepared from traditional open-pollinated maize varieties. Thus, the absence of pyrrolidines may contribute to the distinctive aroma of *broas,* and the high amounts of pyrazines may confer negative characteristics associated with the commercial hybrid maize *broa* analyzed. Sulfur compounds, such as dimethyl trisulfide and methanethiol, were identified as the most likely contributors to the aroma of this ethnic bread. Some volatiles, such as oxathiane, have not been previously reported in similar foods, and their relevance to the overall aroma of foods is currently unknown. The data obtained in this study can be further explored for the purpose of fingerprinting traditional *broas* since some compounds (2-methylpyrazine and 2-butyltetrahydrofuran) were present in significantly higher amounts in the *broa* prepared from the analyzed commercial maize hybrid variety. In conclusion, this work represents the most detailed study on the volatile composition of *broas*. It may contribute to disclosing possible volatiles of other bread and maize-based foods. These findings may have a number of important implications for future practice since better knowledge of the volatile compounds produced along the Maillard reaction may help to achieve the production of foods with sensory characteristics more appreciated by consumers. 

## Figures and Tables

**Table 1:** Volatile characteristics from browning reactions identified in *broas*, including their average peak areas, odor and taste descriptors, and odor thresholds; and examples of foods where the compounds have been detected.

ID	1tR	^2^t_R_	Compound	Odor and Taste Descriptors	CAS	Formula	m/z	SI	LRIc	LRIlit	PA	OT (ppb)	Log10 PA/OT	Foods
**Furans**														
**F1**	95	0.38	2,3-Dihydrofuran	Pungent	1191-99-7	C_4_H_6_O	70, 41, 39, 29	891	600	n/f	3,100,662 ± 2,605,740	n/f	n/a	WRB
**F2**	100	0.40	2-Methylfuran or 3-Methylfuran (TI)	Ethereal, acetone, chocolate n/f	534-22-5 930-27-8	C_5_H_6_O	82, 53, 39, 27	912 900	606	605 611	3,101,036 ± 1,363,010	90,450 n/f	1.5 n/a	P, RSB, TB, WB, WRB RSB, WB
**F3**	130	0.43	2,3-Dihydro-5-methylfuran	n/f	1487-15-6	C_5_H_8_O	43, 84, 39, 27, 69	872	641	n/f	1,630,190 ± 846,164	n/f	n/a	n/i
**F4**	140	0.40	Tetrahydrofuran	Ethereal	109-99-9	C_4_H_8_O	42, 72, 27	942	647	633	15,225,039 ± 4,244,701	7375–177,000	1.9–3.3	WB, WP
**F5**	155	0.47	2-Ethylfuran	Rubbery, pungent, acid, sweet	3208-16-0	C_6_H_8_O	81, 53, 96, 39	975	662	689	2,595,043 ± 621,119	n/f	n/a	P, RS, WB
**F6**	160	0.47	2,5-Dimethylfuran	Chemical, ethereal, meaty, gravy, juicy, bacon	625-86-5	C_6_H_8_O	43, 96, 53, 81, 27, 39	916	667	667	843,893 ± 397,252	n/f	n/a	WRB
**F7**	270	0.58	2-Propylfuran	n/f	4229-91-8	C_7_H_10_O	81, 53, 110, 39, 27	973	780	792	613,397 ± 229,105	n/f	n/a	WB
**F8**	295	0.59	2-Ethyl-5-methylfuran	Fresh, gassy, burnt	1703-52-2	C_7_H_10_O	95, 110, 43	877	803	803	357,713 ± 172,466	n/f	n/a	n/i
**F9**	325	0.60	2,3,5-Trimethylfuran	n/f	10504-04-8	C_7_H_10_O	110, 109, 43, 95, 67	896	819	817	64,938 ± 50,557	n/f	n/a	WB
**F10**	325	3.65	3-Furfural (3-furancarboxaldehyde)	Almond-like	498-60-2	C_5_H_4_O_2_	95, 39, 67, 29	942	820	832	4,539,412 ± 1,121,048	n/f	n/a	GFB, NSB, P, RSB, WB
**F11**	355	0.80	2-Vinyl-5-methylfuran	n/f	10504-13-9	C_7_H_8_O	108, 65, 43, 79, 56, 93	889	835	826	295,635 ± 120,061	n/f	n/a	n/i
**F12**	360	3.43	**Furfural** **(2-furancarboxaldehyde)**	Woody, almond, sweet, fruity, flowery	98-01-1	C_5_H_4_O_2_	96, 39, 29	966	839	843	200,344,626 ± 78,855,174	3000–23,000	3.9–4.8	B, BAG, CWB, ME, GFB, NSB, P, T, TB, TC, S, WB, WSB
**F13**	425	1.70	**Furfuryl alcohol** **(2-furanmethanol)**	Weak, fermented, creamy, caramel	98-00-0	C_5_H_6_O_2_	39, 41, 53, 81, 70, 98	949	872	865	404,124,163 ± 136,284,119	2000	5.3	B, CWB, ME, GFB, NSB, P, RSB, T, TB, TC, TF, TS, WB, WP, WSB
**F14**	465	0.56	**2-*n*-Butyl furan**	Green	4466-24-4	C_8_H_12_O	81, 53, 39, 27	951	892	898	5,012,989 ± 1,768,099	50,800	2.0	B, RS, WB
**F15**	490	1.77	Furfuryl formate	Ethereal	13493-97-5	C_6_H_6_O_3_	81, 53, 39, 44	918	909	902	643,862 ± 233,472	n/f	n/a	WB
**F16**	495	1.77	**2-Acetylfuran** **(1-(2-furanyl)-ethanone)**	Smoky, roasty	1192-62-7	C_6_H_6_O_2_	95, 39, 43, 67	921	912	915	49,074,458 ± 10,718,656	10,000	3.7	B, CWB, ME, GFB, P, RSB, T, TC, TF, WB, WP, WSB
**F17**	555	1.37	1-(2-Furyl)-2-propanone (2-furyl acetone)	Herbal, caramel, fruity, spicy, radish, green, burnt	6975-60-6	C_7_H_8_O_2_	81, 43, 53, 124	915	957	954	2,719,968 ± 2,692,555	n/f	n/a	WB
**F18**	565	1.61	**5-Methyl furfural** **(5-methyl-2-furancarboxaldehyde)**	Almond, sweet, bitter	620-02-0	C_6_H_6_O_2_	110, 53, 39, 43, 81	979	964	966	33,471,223 ± 8,857,491	500	4.8	B, ME, GFB, NSB, P, RSB, TB, WB
**F19**	565	2.98	5-methyl-2-furanmethanol (5-methylfurfuryl alcohol)	Bread-like, honey, sweet	3857-25-8	C_6_H_8_O_2_	95, 112, 43, 69, 53	913	965	966	8,379,336 ± 4,226,245	11.9	5.8	BAG, CWB, WB
**F20**	600	0.53	**2-Pentylfuran**	Butter, green bean, floral, fruity, mushroom, raw nuts	3777-69-3	C_9_H_14_O	81, 53, 39	934	989	993	55,139,336 ± 14,203,476	6	7.0	B, BAG, ME, GFB, MF, MJ, NSB, P, RS, TC, TF, TS, WB, WP, WRB
**F21**	605	1.05	Benzofuran	Styrene, aromatic	271-89-6	C_8_H_6_O	118, 89, 63, 39, 51, 45	892	993	996	470,358 ± 286,276	n/f	n/a	n/i
**F22**	605	1.53	Isomaltol (2-acetyl-3-hydroxyfuran)	Caramel-like	3420-59-5	C_6_H_6_O_3_	111, 126, 43, 55, 84	918	994	989	392,890 ± 417,850	n/f	n/a	n/i
**F23**	610	1.12	Furfuryl acetate (2-furanmethanol acetate)	Sweet, fruity, banana-like, horseradish, ethereal, green	623-17-6	C_7_H_8_O_3_	81, 98, 43, 52, 140	920	997	998	5,202,352 ± 2,702,948	n/f	n/a	WB
**F24**	625	1.21	1-(2-Furanyl)-1-propanone	Fruity	3194-15-8	C_7_H_8_O_2_	95, 39, 45, 74, 67, 57	872	1011	1016	2,589,462 ± 1,364,329	n/f	n/a	WP
**F25**	645	1.22	2-Acetyl-5-methylfuran	Sweet, nutty with a caramel nuance, cocoa-like with a toasted bready nuance	1193-79-9	C_7_H_8_O_2_	109, 124, 43, 53	928	1030	1042	7,472,295 ± 7,428,702	n/f	n/a	n/i
**F26**	655	1.13	2,2′-Bifuran (2-(2-furanyl)furan)	Vegetable, garlic	5905-00-0	C_8_H_6_O_2_	134, 78, 105, 51, 39	893	1039	1047	998,680 ± 662,678	n/f	n/a	WB
**F27**	705	0.50	**Methylpentylfuran**	n/f	-	C_10_H_20_O	95, 152, 43, 67	892	1086	1083	3,099,292 ± 2,322,514	n/f	n/a	B
**F28**	705	0.95	2-Furfurylfuran (2,2′-Methylenebisfuran)	Rich, roasted, aromatic	1197-40-6	C_9_H_8_O_2_	91, 148, 120, 39, 65	863	1087	1086	451,285 ± 232,810	n/f	n/a	n/i
**F29**	710	3.24	5-Formylfurfural (2,5-furandicarboxaldehyde)	n/f	823-82-5	C_6_H_4_O_3_	124, 77	879	1094	1084	5,731,606 ± 2,639,042	n/f	n/a	WB
**F30**	715	0.51	2-Hexylfuran	n/f	3777-70-6	C_10_H_16_O	81, 53, 39, 41, 95, 123	905	1096	1096	463,776 ± 178,720	n/f	n/a	n/i
**F31**	715	0.77	2-Butyl-tetrahydrofuran	n/f	1004-29-1	C_8_H_16_O	71, 41, 55	864	1096	1096	3,919,696 ± 3,110,078	n/f	n/a	n/i
**F32**	715	3.29	Furyl hydroxymethyl ketone (1-(2-furanyl)-2-hydroxyethanone)	n/f	17678-19-2	C_6_H_6_O_3_	95, 39, 126, 29, 67	948	1098	1088	21,691,749 ± 9,667,403	n/f	n/a	n/i
**F33**	805	1.32	1-(5-Methyl-2-furanyl)-2-hydroxyethanone	n/f	-	C_7_H_8_O_3_	109, 56, 69, 43, 140	833	1191	n/f	1,550,219 ± 734,005	n/f	n/a	n/i
**F34**	865	4.97	**2,3-Dihydrobenzofuran**	Musky notes	496-16-2	C_8_H_8_O	120, 91, 65, 51	911	1265	1222	2,807,760 ± 735,738	n/f	n/a	B, BMJ
**F35**	905	1.17	Difurfuryl ether (2,2′-[oxybis(methylene)]*bis*-furan)	Coffee, mushroom-like, nutty, earthy	4437-22-3	C_10_H_10_O_3_	81, 56, 27, 39, 97, 110	944	1308	1305	240,009 ± 95,353	n/f	n/a	n/i
**F36**	935	4.45	Hydroxymethylfurfural (5-(Hydroxymethyl)furfural)	Fatty, buttery, musty, waxy, caramel, herbal, tobacco	67-47-0	C_6_H_6_O_3_	97, 126, 41, 69, 53	907	1325	1266	22,784,687 ± 12,185,059	1,000,000	1.4	P, RSB, WSB
**Furanones**														
**Fo1**	245	3.32	2-Furanone (TI) (2(3*H*)-furanone)	n/f	20825-71-2	C_4_H_4_O_2_	55, 84, 27, 53, 39, 44	873	757	914 (DB-1)	2,079,421 ± 625,998	n/f	n/a	n/i
**Fo2**	305	1.58	Dihydro-2-methyl-3-(2*H*)-furanone (2-methyltetrahydro-3-furanone)	Spicy, rancid, butter	3188-00-9	C_5_H_8_O_2_	43, 72, 100	974	809	812	19,616,932 ± 16,891,970	n/f	n/a	WB, WP
**Fo3**	420	3.11	5-Methyl-2(3*H*)-furanone	Sweet, oily, coconut, tobacco, creamy, vanilla, hay	591-12-8	C_5_H_6_O_2_	55, 98, 43, 27, 70	851	870	869	235,195 ± 69,497	n/f	n/a	n/i
**Fo4**	480	0.83	5-Methyl-5-furfuryl-2(5*H*)-furanone (5-(2-furanylmethyl)-5-methyl-2(5*H*)-furanone) (TI)	n/f	31969-27-4	C_10_H_10_O_3_	81, 53, 39, 69	751	901	n/f	1,263,633 ± 1,567,577	n/f	n/a	WB
**Fo5**	505	4.88	2(5*H*)-Furanone (2,5-dihydrofuranone)	n/f	497-23-4	C_4_H_4_O_2_	55, 84, 27, 29, 39	940	922	918	43,176,086 ± 16,137,613	n/f	n/a	WB, CWB
**Fo6**	540	2.77	5-Methyl-2(5*H*)-furanone	n/f	591-11-7	C_5_H_6_O_2_	69, 41, 39, 98	942	943	938	6,155,177 ± 2,897,005	n/f	n/a	WB
**Fo7**	550	1.31	3-Methyl-2,5-furandione or 2,5-Dimethyl-3(2*H*)-furanone (TI)	n/f n/f	616-02-4 14400-67-0	C_5_H_4_O_3_	39, 68, 40, 28, 53, 112	706 926	953	949 924 (DB-1)	1,818,359 ± 629,312	n/f	n/a	WB n/i
**Fo8**	555	1.93	γ-Valerolactone (dihydro-5-methyl-2(3*H*)-furanone)	Herbal, sweet, warm, tobacco, cocoa, woody, coconut	108-29-2	C_5_H_8_O_2_	56, 85, 41, 43	958	956	956	3,864,613 ± 1,759,554	n/f	n/a	WB, CWB
**Fo9**	555	1.91	5,5-Dimethyl-2(5*H*)-furanone	n/f	20019-64-1	C_6_H_8_O_2_	97, 69, 43, 54, 26, 112	891	957	958	250,199 ± 71,863	n/f	n/a	n/i
**Fo10**	560	1.90	Dihydro-3-methyl-2(3*H*)-furanone or Dihydro-4-methyl-2(3*H*)-furanone (3-methylbutyrolactone) (TI)	n/f n/f	1679-47-6 1679-49-8	C_5_H_8_O_2_	41, 56, 27, 100	910 917	961	958 919	2,130,675 ± 842,009	n/f n/f	n/a n/a	n/i n/i
**Fo11**	565	1.84	Dihydro-3-methyl-2(3*H*)-furanone or Dihydro-4-methyl-2(3*H*)-furanone (3-methylbutyrolactone) (TI)	n/f n/f	1679-47-6 1679-49-8	C_5_H_8_O_2_	56, 85, 41, 43, 100	895 891	964	958 919	502,907 ± 283,902	n/f n/f	n/a n/a	n/ n/i
**Fo12**	570	1.87	n/i	n/a	-	C_6_H_8_O_2_	97, 69, 43, 26, 54	n/a	968	n/a	183,534 ± 115,610	n/a	n/a	n/a
**Fo13**	575	1.24	5-Ethyl-(3*H*)-furan-2-one (2-ethylbutenolide)	Spicy	2313-01-1	C_6_H_8_O_2_	55, 112, 83, 97	910	971	954	612,612 ± 196,297	n/f	n/a	WB
**Fo14**	615	1.64	2,5-Dihydro-3,5-dimethyl 2-furanone	n/f	5584-69-0	C_6_H_8_O_2_	69, 41, 115, 97	884	1002	993	973,653 ± 442,005	n/f	n/a	n/i
**Fo15**	655	1.95	5-Ethyl-2(5*H*)-Furanone (TI)	Spicy	2407-43-4	C_6_H_8_O_2_	28, 83, 18, 55, 44	897	1040	984 (DB-1)	5,650,222 ± 2,621,703	n/f	n/a	WB
**Fo16**	655	1.79	3,4-Dimethyl-2,5-furandione (2,3-dimethyl maleic anhydride)	n/f	766-39-2	C_6_H_6_O_3_	39, 54, 82, 126	882	1040	1038	3,343,389 ± 1,471,465	n/f	n/a	n/i
**Fo17**	665	4.90	***R*-Pantolactone**	Cotton candy, licorice, smoky, toasted bread	599-04-2	C_6_H_10_O_3_	71, 43, 29, 57	951	1047	1043	2,268,814 ± 1,125,826	50	4.7	MF
**Fo18**	670	2.82	4-Methyl-2(5*H*)-furanone	n/f	6124-79-4	C_5_H_6_O_2_	69, 41, 39, 98	924	1055	n/f	2,855,095 ± 885,123	n/f	n/a	n/i
**Fo19**	675	1.45	**γ-*N*-Caprolactone** **(γ-hexalactone,** **5-ethyldihydro-2(3*H*)-furanone)**	Coumarin-like, sweet	695-06-7	C_6_H_10_O_2_	85, 42, 56, 70, 114	942	1059	1058	15,427,139 ± 6,696,866	50	5.5	B, CWB
**Fo20**	705	4.02	Furaneol (2,5-dimethyl-4-hydroxy-3(2*H*)-furanone)	Caramel, strawberry	3658-77-3	C_6_H_8_O_3_	43, 57, 128, 85	918	1090	1090	3,801,345 ± 2,827,164	60	4.8	WB, BAG, CWB, GFB, NSB, P, RS, TC
**Fo21**	765	3.00	Solerone (TI) (5-acetyldihydro-2(3*H*)-furanone)	n/f	29393-32-6	C_6_H_8_O_3_	85, 29, 43, 57, 128	937	1151	1299 (SE-54)	2,040,510 ± 929,704	n/f	n/a	n/i
**Fo22**	775	1.26	γ-Heptalactone (dihydro-5-propyl-2(3*H*)-furanone)	Sweet, coconut, nutty, caramel, creamy, milky, tobacco	105-21-5	C_7_H_12_O_2_	85, 29, 56, 41	948	1159	1163	833,364 ± 298,059	499	3.2	n/i
**Fo23**	870	1.17	γ-Octalactone (5-butyldihydro-2(3*H*)-furanone)	Sweet, coconut, waxy, creamy, milky, soapy, fruity	104-50-7	C_8_H_14_O_2_	85, 41, 56, 100	955	1266	1264	1,318,130 ± 558,277	8	5.2	CWB, TC
**Fo24**	875	0.89	5-Pentyl-2(3*H*)-furanone (3-nonen-4-olide)	Tropical, fruity, milky, dairy	51352-68-2	C_9_H_14_O_2_	98, 111, 55, 83, 70, 154	840	1272	1273	3,499,672 ± 1,292,082	n/f	n/a	n/i
**Fo25**	940	1.29	5-Pentyl-2(5*H*)-furanone (4-hydroxy-2-nonenoic acid lactone)	Minty, fruity	21963-26-8	C_9_H_14_O_2_	29, 28, 45, 57, 100, 113, 126, 85, 72	823	1352	1358	10,012,186 ± 4,179,765	n/f	n/a	n/i
**Fo26**	955	1.10	γ-Nonalactone (dihydro-5-pentyl-2(3*H*)-furanone)	Coconut-like, sweet, fruity	104-61-0	C_9_H_16_O_2_	85, 114, 41, 55, 99, 137	946	1370	1363	9,304,844 ± 4,182,379	9.7–27	5.5–6.0	B, BMJ, CWB, MJ, WSB
**Pyrans**														
**Pn1**	240	0.53	3,4-Dihydro-6-methyl-2*H*-pyran	n/f	16015-11-5	C_6_H_10_O	43, 55, 98, 83	913	749	n/f	134,400 ± 30,229	n/f	n/a	n/i
**Pn2**	675	1.50	5,6-Dihydro-2*H*-pyran-2-carboxaldehyde or 3,4-Dihydro-2*H*-pyran-2-carboxaldehyde (TI)	n/f n/f	53897-26-0 100-73-2	C_6_H_8_O_2_	83, 55, 29, 112, 39	867 860	1059	n/f 853 (OV-101)	1,444,173 ± 653,900	n/f n/f	n/a n/a	n/i
**Pn3**	915	1.02	2-(1-Butenyl)-tetrahydropyran	n/f	95652-24-7	C_9_H_16_O	111, 140, 83, 98, 125	801	1320	n/f	129,456 ± 54,493	n/f	n/a	n/i
**Pyranones**														
**Po1**	450	1.47	Dihydro-2*H*-pyran-3(4*H*)-one (TI)	n/f	23462-75-1	C_5_H_8_O_2_	42, 27, 71, 55	947	885	1439 (HP-Wax)	54,028 ± 25,761	n/f	n/a	n/i
**Po2**	585	3.05	2*H*-Pyran-2-one (α-pyrone)	Herbal	504-31-4	C_5_H_4_O_2_	39, 68, 96	877	980	978	34,118 ± 13,808	n/f	n/a	WP
**Po3**	650	1.58	n/i	n/a	n/a	n/a	68, 39, 98, 53	801 756	1035	n/a	5,592,787 ± 4,970,053	n/a n/a	n/a n/a	n/i
**Po4**	655	2.74	5,6-Dihydro-2*H*-pyran-2-one (TI)	n/f	3393-45-1	C_5_H_6_O_2_	68, 39, 98, 53	929	1041	1838 (DB-Wax)	1,621,497 ± 558,094	n/f	n/a	n/i
**Po5**	675	2.03	δ-Valerolactone (TI) (tetrahydro-2*H*-pyran-2-one)	n/f	542-28-9	C_5_H_8_O_2_	42, 41, 27, 56, 100, 70	947	1059	965	916,387 ± 142,550	n/f	n/a	n/i
**Po6**	715	1.60	δ-Hexalactone (tetrahydro-6-methyl-2*H*-pyran-2-one, δ-caprolactone)	Creamy, fruity, coconut, spicy	823-22-3	C_6_H_10_O_2_	42, 70, 55, 99	944	1097	1084	840,593 ± 226,566	n/f	n/a	WB
**Po7**	740	2.64	**Maltol** **(3-hydroxy-2-methyl-4*H*-pyran-4-one)**	Warmy-fruity, caramel-sweet	118-71-8	C_6_H_6_O_3_	126, 71, 43, 55, 97	974	1124	1113	117,672,149 ± 40,223,031	2500	4.7	B, P, TB, TC, WB
**Po8**	795	0.20	**3-Hydroxy-2,3-dihydromaltol** **(2,3-dihydro-3,5-dihydroxy-6-methyl-4*H*-pyran-4-one)**	Caramelized	28564-83-2	C_6_H_8_O_4_	43, 144, 101, 73, 55	972	1179	1144	29,961,549 ± 16,035,481	n/f	n/a	B, P, WB
**Po9**	975	2.40	2-Hydroxy-3-methyl-4*H*-pyran-4-one	n/f	61892-88-4	C_6_H_6_O_3_	126, 71, 43, 55, 97	818	1397	n/f	37,421 ± 25,172	n/f	n/a	n/i
**Pyrazines**														
**Pz1**	190	1.25	Pyrazine	Roasted	290-37-9	C_4_H_4_N_2_	80, 26, 53	960	704	739	1,631,354 ± 797,679	180,000	1.0	WB, ME, GFB, NSB, P, TC, TS, WP
**Pz2**	345	1.34	**2-Methylpyrazine**	Roasted, burnt, sweet	109-08-0	C_5_H_6_N_2_	94, 67, 39, 26, 53	974	830	840	15,773,787 ± 11,169,354	60–105,000	2.2–5.4	B, WB, BAG, ME, GFB, NSB, P, T, TC, TS, WFB, WP, WRB
**Pz3**	495	0.90	2,6-Dimethylpyrazine	Roasted	108-50-9	C_6_H_8_N_2_	42, 108, 39, 40, 28, 18	972	912	915	23,375,159 ± 12,540,414	200–9000	3.4–5.1	WB, CWB, GFB, NSB, P, T, TC, WFB, WP, WRB
**Pz4**	500	0.91	2-Ethylpyrazine	Popcorn, nutty	13925-00-3	C_6_H_8_N_2_	107, 108, 80, 53, 39, 28	926	915	915	2,149,106 ± 1,864,070	6000–22,000	2.0–2.6	WB, ME, GFB, NSB, P, RSB, T, TC, TS, WRB
**Pz5**	505	0.93	2,3-Dimethylpyrazine	Popcorn, roasted	5910-89-4	C_6_H_8_N_2_	108, 67, 42	929	919	919	2,413,964 ± 1,493,485	2500–35,000	1.8–3.0	WB, ME, GFB, NSB, P, T, TC, TS
**Pz6**	615	0.75	2-Ethyl-3-methylpyrazine or 2,3,5-trimethylpyrazine (TI)	Potato-like, earthy Nutty, roasted, sweet	15707-23-0 14667-55-1	C_7_H_10_N_2_	42, 122, 81, 39	954 936	1001	1001 1005	5,256,433 ± 2,326,845	400–1800 130	4.6 3.5–4.1	WB, ME, GFB, NSB, P, RSB, TC, WFB, WP, WRB WB, BAG, ME, GFB, TC
**Pz7**	630	0.90	2-Ethenyl-6-methylpyrazine (2-methyl-6-vinylpyrazine)	Coffee	13925-09-2	C_7_H_8_N_2_	120, 52, 39, 94	834	1015	1023	918,205 ± 550,329	n/f	n/a	WB, ME, P, T, WP
**Pz8**	640	1.35	Acetylpyrazine	Biscuit, cracker-like, crust-like, sweet, roasted	22047-25-2	C_6_H_6_N_2_O	43, 52, 80, 122, 94, 28, 15	965	1025	1031	418,788 ± 223,595	62	3.8	WB, GFB, NSB, RSB, WRB
**Pz9**	700	0.65	2-Ethyl-3,6-dimethylpyrazine (3-ethyl-2,5-dimethyl-pyrazine) or 2-Ethyl-3,5-dimethyl-pyrazine (3-ethyl-2,6-dimethylpyrazine)	Potato, cocoa, roasted, nutty Burnt, roasted, nutty, coffee, caramel, cocoa, maize	13360-65-1 13925-07-0	C_8_H_12_N_2_	135, 136, 42, 39, 56, 108	918 885	1082	1082 1082	749,792 ± 503,023	8.6 0.04–1	4.9 5.9–7.3	WB, ME, P, RSB WB, ME, P, T, TC, WRB, WSB
**Pz10**	740	1.07	2-Acetyl-3-methylpyrazine	Nutty, roasted, hazelnut, corn chip, caramel, potato chip	23787-80-6	C_7_H_8_N_2_O	43, 93, 136, 94, 42, 67, 52	864	1122	1128	207,115 ± 107,124	n/f	n/a	n/i
**Pz11**	760	0.83	5*H*-5-Methyl-6,7-dihydrocyclopentapyrazine	Earthy, baked potato, sweet, roasted, corn with savory	23747-48-0	C_8_H_10_N_2_	119, 134	907	1143	1149	511,503 ± 176,711	n/f	n/a	n/i
**Pz12**	875	1.40	2-(2′-Furyl)-pyrazine (TI) (2-(furan-2-yl)pyrazine)	n/f	32736-95-1	C_8_H_6_N_2_O	146, 93, 63, 38, 39	816	1272	1255 (DB-1)	293,650 ± 139,715	n/f	n/a	n/i
**Pyridines and Pyrimidines**														
**Pd1**	350	1.05	2-Methylpyridine	Sweat, astringent, hazelnut, nutty	109-06-8	C_6_H_7_N	93, 66, 39, 78, 51	958	832	824	349,470 ± 195,878	n/f	n/a	n/i
**Pd2**	420	0.98	4-Methylpyrimidine	n/f	3438-46-8	C_5_H_6_N_2_	94, 40, 53, 67, 79	802	869	853	9105 ± 6947	n/f	n/a	n/i
**Pd3**	460	0.79	2,6-Dimethylpyridine (2,6-lutidine)	Nutty, ammoniacal, woody, bready, cocoa, coffee, musty	108-48-5	C_7_H_9_N	107, 106, 39, 66, 79, 93	924	890	890	306,301 ± 109,246	n/f	n/a	n/i
**Pd4**	490	0.79	2-Ethylpyridine (TI)	Green, grassy	100-71-0	C_7_H_9_N	106, 107, 79, 52, 66, 39	702	908	906	135,706 ± 64,908	n/f	n/a	T
**Pd5**	550	0.83	2,3-Dimethylpyridine (2,3-lutidine)	Coffee, caramel	583-61-9	C_7_H_9_N	107, 106, 39, 66, 79, 92	937	952	952	29,778 ± 9134	n/f	n/a	n/i
**Pd6**	605	0.71	2,4,6-Trimethylpyridine	Aromatic odor	108-75-8	C_8_H_11_N	121, 79, 39, 106	842	993	993	167,322 ± 96,358	n/f	n/a	n/i
**Pd7**	650	1.18	2-Acetylpyridine (1-(2-pyridinyl)-ethanone)	Biscuit-like, toasted, cracker-like, crust-like, roasted	1122-62-9	C_7_H_7_NO	79, 121, 93, 43, 51	939	1035	1050	1,141,684 ± 1,282,175	19	4.8	ME, P, T, TC
**Pd8**	810	1.13	1-Acetyl-1,2,3,4-tetrahydropyridine	Nutty	19615-27-1	C_7_H_11_NO	85, 125, 83, 68, 43, 54	882	1196	1189	1,504,556 ± 534,232	n/f	n/a	n/i
**Pd9**	815	0.65	2-Pentylpyridine (2-propylpyridine)	Green, fatty, roasted, tobacco, nutty	622-39-9	C_8_H_11_N	93, 106, 120, 79, 65, 39	850	1201	1202	89,596 ± 34,399	0.6	5.2	n/i
**Pyrroles**														
**Py1**	315	1.04	1-Ethyl-1*H*-pyrrole	Burnt	617-92-5	C_6_H_9_N	80, 95, 67, 39, 27, 53	879	814	815	68,416 ± 27,880	n/f	n/a	ME
**Py2**	615	1.48	*N*-Methyl-2-formylpyrrole (1-methyl-1*H*-pyrrole-2-carboxaldehyde)	Roasted, nutty	1192-58-1	C_6_H_7_NO	109, 108, 53, 80, 39	920	1004	1010	848,932 ± 340,434	37	4.4	P
**Py3**	665	1.14	1-Ethyl-2-formyl-1*H*-pyrrole (1-ethyl-1*H*-pyrrole-2-carboxaldehyde)	Burnt, roasted, smoky	2167-14-8	C_7_H_9_NO	123, 94, 39, 108, 66, 53, 80	816	1047	1046	664,513 ± 343,483	n/f	n/a	ME
**Py4**	665	0.14	2-Formyl-1*H*-pyrrole (1*H*-pyrrole-2-carboxaldehyde)	Musty	1003-29-8	C_5_H_5_NO	95, 94, 66, 39	949	1048	1047	2,142,059 ± 1,055,032	n/f	n/a	ME, P, WB
**Py5**	670	1.38	1-Methyl-2-pyrrolidinone	Fishlike	872-50-4	C_5_H_9_NO	44, 42, 99, 98, 28	849	1057	1046	331,816 ± 211,791	n/f	n/a	n/i
**Py6**	690	3.46	**2-Acetylpyrrole** **(1-(1*H*-pyrrol-2-yl)-ethanone)**	Musty	1072-83-9	C_6_H_7_NO	94, 109, 66, 39, 43, 53	954	1076	1069	21,389,485 ± 7,698,915	170,000	2.1	B, BAG, CWB, GFB, NSB, P, TC, WB
**Py7**	735	1.14	1-Ethyl-2-pyrrolidinone (TI)	Slight amine	2687-91-4	C_6_H_11_NO	98, 113, 70, 41, 28	907	1117	1856 (FFAP)	209,715 ± 147,230	n/f	n/a	n/i
**Py8**	740	1.42	Ethyl pyrrole 1-acetate (TI)	n/f	5145-67-5	C_8_H_11_NO_2_	80, 153, 53, 57, 71	718	1123	n/f	995,860 ± 1,013,831	n/f	n/a	n/i
**Py9**	800	1.20	***N*-Furfurylpyrrole** **(1-(2-furanylmethyl)-1*H*-pyrrole)**	Vegetable, plastic, waxy, fruity, cereal, bready, potato	1438-94-4	C_9_H_9_NO	81, 147, 53, 27, 39	955	1186	1179	775,590 ± 358,385	100	3.9	B, ME, P, WB, WRB
**Py10**	910	4.10	**Indole** **(benzopyrrole)**	Animal, naphthyl, fecal, pungent, musty; in low concentrations: powerful floral notes and pleasant radiation	120-72-9	C_8_H_7_N	117, 90, 63, 39, 50	954	1318	1295	6,532,212 ± 1,981,606	140	4.7	B, WB
**Py11**	980	1.18	5-Acetyl-2,3-dihydro-1*H*-pyrrolizine (TI)	Amine, grass, hay, smoky	55041-85-5	C_9_H_11_NO	134, 149, 106, 79, 51	910	1402	1382 (DB-1)	807,263 ± 424,045	n/f	n/a	P
**Py12**	985	3.01	Skatole (3-methyl-1*H*-indole)	Animal, fecal, warm, sweet, over-ripe fruit; in low concentrations: may give a note of ‘overmature flower’	83-34-1	C_9_H_9_N	130, 131, 77, 51, 65, 103, 39	940	1411	1410	50,749 ± 33,736	0.2	5.4	P
**Py13**	1000	1.49	1-Furfuryl-2-formyl pyrrole (1-methyl-1*H*-pyrrole-2-carboxaldehyde)	Slightly burnt taste, acid n/f	19377-82-3	C_10_H_9_NO_2_	81, 175, 53, 39, 147	851	1429	1384	284,459 ± 165,711	97	3.5	P, WRB
**Oxazoles**														
**Ox1**	235	0.93	4,5-Dimethyloxazole	n/f	7064-40-6	C_5_H_7_NO	97, 43, 55	821	745	750	29,777 ± 23,570	n/f	n/a	n/i
**Ox2**	410	0.77	Trimethyloxazole	Nutty, roasted, shellfish, mustard burnt, oily, mushroom	20662-84-4	C_6_H_9_NO	111, 43, 68, 55	868	864	863	109,136 ± 176,271	n/f	n/a	WP
**Ox3**	735	1.34	Benzoxazole (TI)	n/f	273-53-0	C_7_H_5_NO	119, 64, 91	853	1117	1067 (DB-1)	182,454 ± 98,420	n/f	n/a	n/i
**Thiazoles**														
**Tz1**	195	1.49	Thiazole	Fishy, nutty, meaty	288-47-1	C_3_H_3_NS	85, 58, 45	967	704	694	294,112 ± 230,369	n/f	n/a	WB
**Tz2**	635	1.46	2-Acetylthiazole	Roasty	24295-03-2	C_5_H_5_NOS	43, 127, 99, 58, 85	944	1020	1018	1,155,525 ± 300,637	10	5.1	WB, ME
**Tz3**	840	1.45	Benzothiazole	Sulfurous	95-16-9	C_7_H_5_NS	135, 108, 69, 45	956	1231	1231	1,124,234 ± 601,368	80	4.1	WB
**Thiophenes**														
**Tp1**	235	0.72	3-Methylthiophene	Fatty, winey	616-44-4	C_5_H_6_S	97, 98, 45	968	744	770	22,399 ± 9459	n/f	n/a	n/i
**Tp2**	600	1.97	3-Thiophenecarboxaldehyde	n/f	498-62-4	C_5_H_4_OS	111, 112, 83, 39	847	990	1003	252,769 ± 141,357	n/f	n/a	n/i
**Tp3**	610	1.98	2-Thiophenecarboxaldehyde (2-formylthiophene, thenaldehyde)	Sulfurous, almond, bitter, cherry	98-03-3	C_5_H_4_OS	111, 112, 83, 58	952	998	1001	762,146 ± 260,839	n/f	n/a	ME
**Tp4**	705	1.31	2-Formyl-3-methylthiophene (3-methyl-2-thiophenecarboxaldehyde)	Saffron, camphoreous	5834-16-2	C_6_H_6_OS	125, 126, 97, 45	845	1087	1109	123,821 ± 40,906	n/f	n/a	n/i
**Tp5**	705	1.56	2-Acetylthiophene (1-(2-thienyl)-ethanone)	Sulfurous, nutty, hazelnut, onion	88-15-3	C_6_H_6_OS	111, 126	829	1087	1085	70,861 ± 22,816	n/f	n/a	WB
**Tp6**	745	1.48	2-Formyl-5-methylthiophene (TI) (5-methyl-2-thiophenecarboxaldehyde)	Rancid, fatty, grass	13679-70-4	C_6_H_6_OS	125, 126, 97, 45	760	1128	1124	90,000 ± 35,587	n/f	n/a	WP, ME
**Other sulfur-containing compounds**														
**S1**	70	0.32	Methanethiol	Rotting cabbage; in low concentrations: tropical fruit, may contribute for the aroma of sweet maize	74-93-1	CH_4_S	47, 45, 48	991	575	<500	6,115,559 ± 1,459,463	0.04–82	4.9–8.2	WB, CB
**S2**	80	0.35	Dimethyl sulfide	Cabbage-like, in low concentrations: canned maize	75-18-3	C_2_H_6_S	47, 62, 35	978	585	565	1,689,726 ± 724,639	0.3–1	6.2–6.8	WB, CB, P, RS
**S3**	195	0.69	Dimethyl disulfide	Garlic; in low concentrations: contributes to maize flavor	624-92-0	C_2_H_6_S_2_	94, 45, 79, 61	973	703	718	9,175,781 ± 4,840,433	12	5.9	WB, CB, ME, GFB, P, RS, TC, WP
**S4**	220	1.56	1-Methylthiopropane (TI) (methyl propyl sulfide)	Alliceous, creamy, green, leek	3877-15-4	C_4_H_10_S	61, 90, 48	714	730	715	100,912 ± 2,3581	n/f	n/a	n/i
**S5**	375	1.58	Methylthio-2-propanone (acetonyl methyl sulfide)	Melon, cabbage, garlic	14109-72-9	C_4_H_8_OS	43, 61, 104	923	846	863	17,904 ± 16,085	n/f	n/a	n/i
**S6**	480	1.10	1,4-Oxathiane, or 1,3-Oxathiane, or 1,2-Oxathiane (thioxane) (TI)	Characteristic n/f Green, grassy, leafy, cortex, foliage, aromatic, vegetable, floral, juicy mango, tropical	15980-15-1 646-12-8 57917-36-9	C_4_H_8_OS	46, 104, 74, 61	811 863 n/a	901	885 n/f n/f	57,599 ± 47,389	n/f n/f n/f	n/a n/a n/a	n/i n/i n/i
**S7**	485	1.52	Methional (3-methylthiopropanal)	Boiled-potato, cooked-potato, malty, waxy	3268-49-3	C_4_H_8_OS	48, 104, 61, 76	930	905	903	806,522 ± 660,313	0.2	6.6	WB, CB, CWB, NSB, P, T, TC, TS, WP
**S8**	565	0.85	Dimethyl trisulfide	Cabbage-like, in low concentrations: tropical fruit/grapefruit	3658-80-8	C_2_H_6_S_3_	126, 79, 45, 111	947	964	964	2,195,136 ± 1,508,373	0.01	8.3	WB, ME, GFB, P, TC, WFB

**ID**: Peak identification;**^1^t_R_**: retention time for the first dimension (seconds); **^2^t_R_**: retention time for the second dimension (seconds); **CAS**: Chemical Abstracts Service registry number; ***m/z***: mass-to-charge ratio (ions are ordered according to their decreasing intensities); **SI**: similarity index (‰); **LRI_c_**: linear retention index obtained through the modulated chromatogram; **LRI_lit_**: linear retention index reported in the literature for Equity-5 column or equivalents [27,34,35,36,37,38,39,40,41,42]; **OT**: Odor threshold values in water [16,32,43,44,45,46,47,48,49,50]; **PA**: average peak area (*n* = 12); **PA/OT:** ratio between PA and OT; **TI**: tentatively identified; **n/a**: not applicable; **n/f**: not found; **n/i**: not identified. Aroma and taste descriptors were obtained from the literature [5,6,13,24,41,42,49,51,52,53,54,55]. Abbreviations used for other maize-based foods or breads where the compounds have been detected [5,6,7,10,16,18,24,27,31,32,33,40,43,49,56,57,58,59,60,61,62]: **MF**: maize flour; **B**: *broa* (compounds highlighted in bold); **WB**: wheat bread; **WSB**: wheat sourdough bread; **WRB**: wheat-rye bread; **RSB**: rye sourdough bread; **GFB**: gluten-free bread; **MSB**: maize starch bread; **CWB**: Chinese white bread; **BAG**: bagels; **WFB**: whole *fino* bread; **TB**: triticale bread; **CB**: crisp bread; **ME**: maize extrudates; **P**: popcorn; **T**: maize tortilla; **TC**: tortilla chips; **TS**: taco shell; **WP**: maize meal extruded product with whey protein; **MJ**: maize juice; **BMJ**: boiled maize juice; **RS**: rye sourdough; **TF**: tortilla flour.

## Data Availability

The data supporting the findings of this study are available within the article and its Supplementary Material.

## Supplementary Materials

The following supporting information can be downloaded at https://www.mdpi.com/article/10.3390/molecules27092728/s1; Figure S1: Visual representation of the volatile baking compounds reported in the literature for several bread and maize-based foods (nodes), Figure S2: Picture of *broas*, Figure S3: Electron ionization mass of Pd8 (1-acetyl-1,2,3,4-tetrahydropyridine) and mass spectra of 1-acetyl-1,2,3,4-tetrahydropyridine and 2-acetyl-1,4,5,6-tetrahydropyridine from the Wiley library, Figure S4: Heatmap and hierarchical cluster analysis representation of the 128 volatiles identified in broas. The content of each metabolite is illustrated through a chromatic scale, Table S1: Maize flours and *broas* identification and description., Table S2: Average total peak areas and % of chromatogram area for the families of chemical compounds studied in *broas*, Table S3: Peak areas obtained for each sample (average of duplicates) and considered for the statistical analysis, Tables S4 and S5: Spearman correlation coefficients among the 128 studied *broas* volatile compounds.

## Sample Availability

Not available.
