# Supplementary material for: Comprehensive Two-Dimensional Gas Chromatography as a Powerful Strategy for the Exploration of Broas Volatile Composition
Source: Molecules. 2022 Apr 23;27(9):2728. doi: 10.3390/molecules27092728 (PMC9102332; doi:10.3390/molecules27092728)
Supplement: Supplementary file 1 [file molecules-27-02728-s001.zip › molecules-1676448-supplementary published/Supplementary Files.pdf]

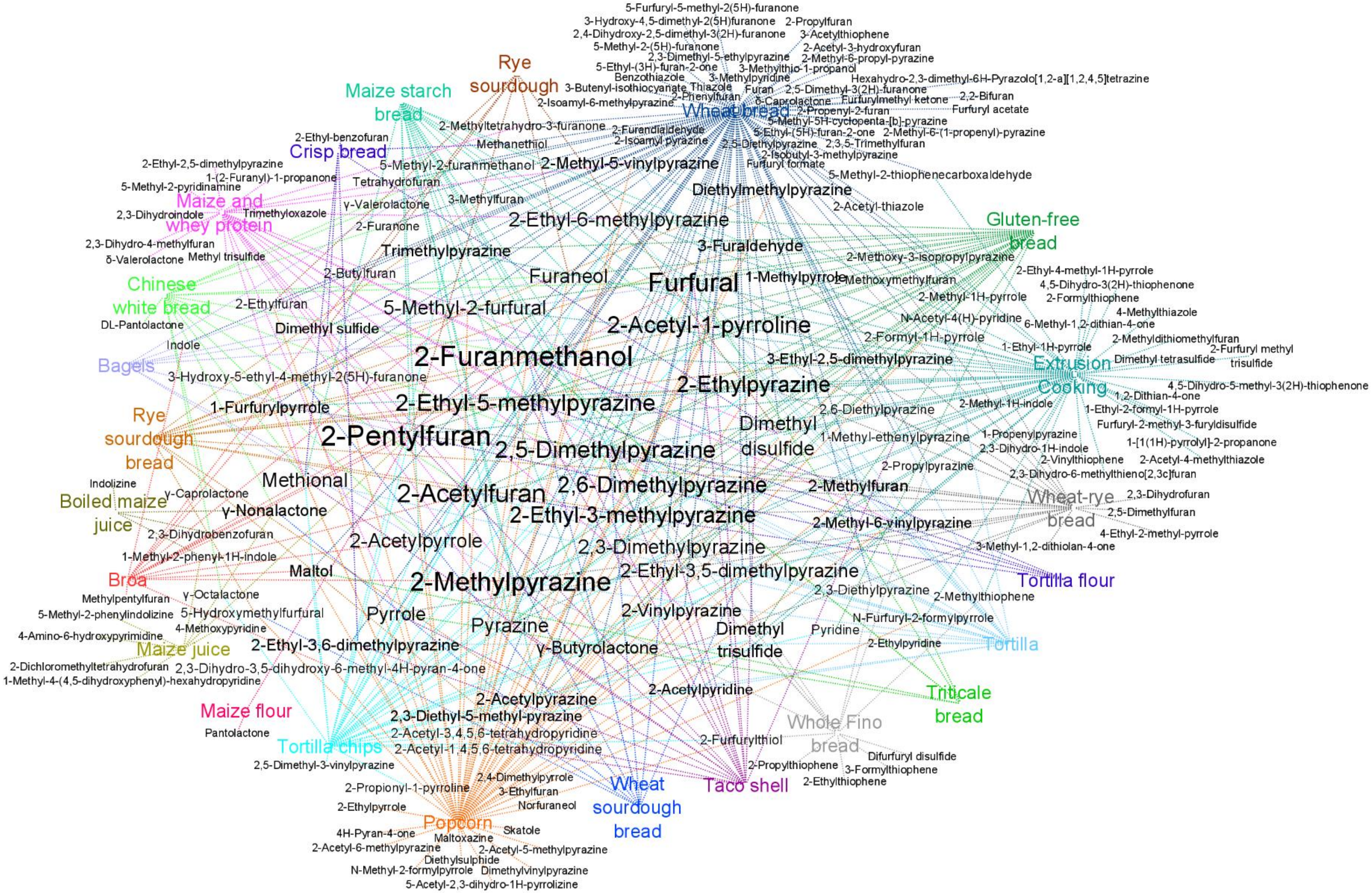

Figure S1. Visual representation of the baking volatile compounds reported in the literature [5–7,10,16,18,24,27,31–33,40,43,49,56–62] for several breads and maize-based foods (nodes). The volatiles more frequently reported (in different foods) are represented with larger font sizes.

Table S1. Maize flours and *broas* identification and description.

Flour	*Broa*	Variety	Kernel Color	Description	Origin
F1	B1	**SinPre**	white	Synthetic open pollinated variety, from the cross of 12 divergent original maize populations developed as an experimental higher-quality cultivar with increased precocity.	VASO participatory maize breeding program [4]
F2	B2	**Aljezudo**	yellow	Flint-type FAO 300. Hybrid open pollinated variety: from the cross made around the years 2000–2005 between two historical populations, Aljezur × Amiudo.	VASO participatory maize breeding program [4]
F3	B3	**Bastos**	white	Early flint open pollinated variety.	VASO participatory maize breeding program [4]
F4	B4	**Amiúdo**	yellow	Early flint-type FAO 200, open pollinated variety adapted to stress conditions (soils with low pH, water stress and aluminum toxicity)	VASO participatory maize breeding program [4]
F5	B5	**Broa-213**	yellow	Early intermediate type, traditional farmer open pollinated variety.	Collected from the farmer in the 2005 expedition to the Central Northern region of Portugal [1]
F6	B6	**Pigarro**	white	Flint-type FAO 300, open pollinated variety, with strong fasciation expression, used in the best soils for human consumption	VASO participatory maize breeding program [4]
F7	B7	**Algarro**	yellow	Early flint-type. Hybrid open pollinated variety: from the cross made around the years 2000–2005 between two historical populations, Aljezur × Pigarro	VASO participatory maize breeding program [4]
F8	B8	**Castro Verde**	yellow	Late flint-type FAO 600, open pollination variety, with big kernel row number and large ear size	VASO participatory maize breeding program [4]
F9	B9	**Verdeal de Aperrela**	white	Late flint-type FAO 600, open pollinated variety, used for bread making	VASO participatory maize breeding program [4]
F10	B10	**Fandango**	yellow	Synthetic open pollinated variety, dent-type FAO 600, big kernel row number and large ear size	VASO participatory maize breeding program [4]
F11	B11	**Broa-57**	white	Early flint-type traditional farmer open pollinated variety	Collected from the farmer in the 2005 expedition to the Central Northern region of Portugal [1]
F12	B12	**Commercial**	white	Nacional Type 175, wholegrain flour (from hybrid maize variety)	Obtained already milled from a bakery

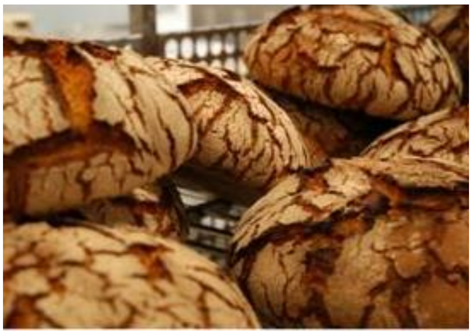

Figure S2. Picture of *broas*. Photo courtesy of Carla Brites.

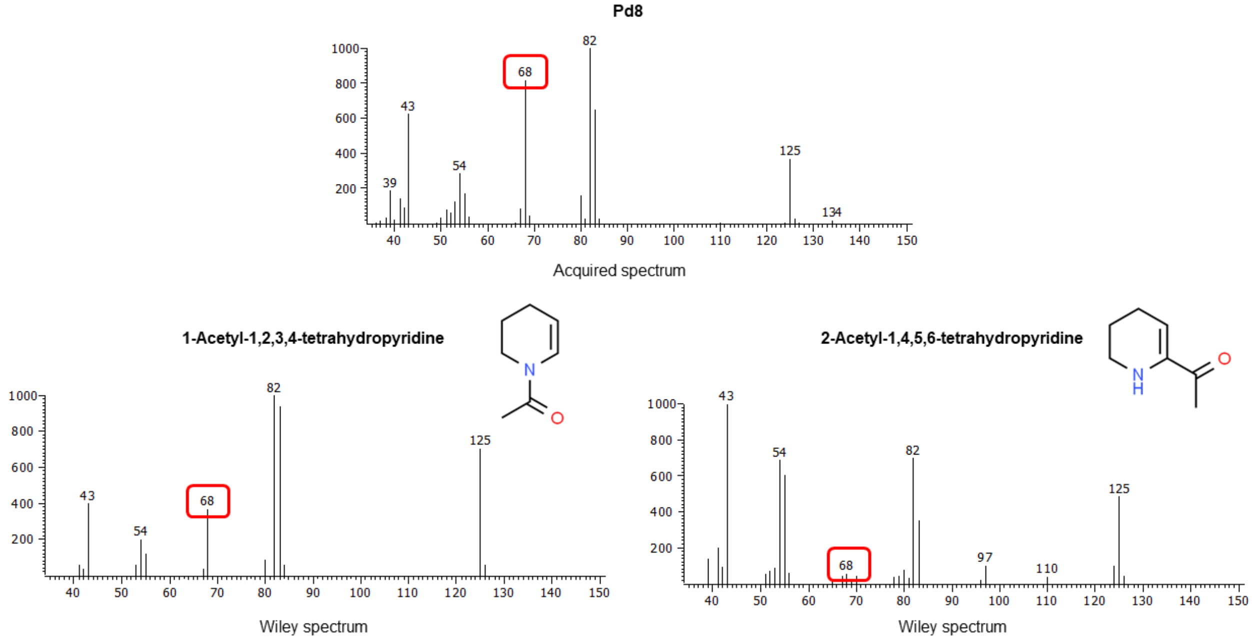

Figure S3. Electron ionization mass of Pd8 (1-acetyl-1,2,3,4-tetrahydropyridine) and mass spectra of 1-acetyl-1,2,3,4-tetrahydropyridine and 2-acetyl-1,4,5,6-tetrahydropyridine from the Wiley library.

Table S2. Average total peak areas and % of chromatogram area for the families of chemical compounds studied in *broas*. Mean values within rows with no letters (a–f or A–G) in common are significantly different ($p < 0.05$).

Broa	Furans	Furanones	Pyrans	Pyranones	Pyrazines	Pyridines	Pyrroles	Oxazoles	Thiazoles	Thiophenes	Sulfides
**B1**	763602279 ± 58932402^{abcd}	123703256 ± 10093713^{b}	1222564 ± 221783^{ab}	127142718 ± 31923812^{ab}	54598819 ± 1318758^{cd}	1899809 ± 436122^{a}	30763217 ± 6724318^{abc}	245164 ± 3953^{ab}	4025450 ± 16575^{c}	1310212 ± 237537^{abcde}	15195450 ± 4374617^{abc}
%	**68 ± 0.1^{AB}**	**11.0 ± 0.03^{BCD}**	**0.11 ± 0.028^{ABCD}**	**11.2 ± 2.0^{AB}**	**4.9 ± 0.50^{BC}**	**0.17 ± 0.026^{A}**	**2.8 ± 0.82^{AB}**	**0.022 ± 0.001^{BC}**	**0.36 ± 0.027^{G}**	**0.12 ± 0.030^{AB}**	**1.4 ± 0.50^{AB}**
**B2**	572947817 ± 769839^{a}	55576697 ± 3171058^{a}	1078252 ± 60845^{a}	128987045 ± 14171961^{ab}	21759529 ± 1018204^{a}	1685579 ± 150533^{a}	18855416 ± 228942^{a}	22441 ± 8866^{a}	1979246 ± 214345^{ab}	916267 ± 184068^{ab}	9535746 ± 788673^{a}
%	**70 ± 1.6^{AB}**	**6.8 ± 0.24^{A}**	**0.13 ± 0.005^{BCDE}**	**15.8 ± 1.4^{B}**	**2.7 ± 0.07^{A}**	**0.21 ± 0.014^{AB}**	**2.3 ± 0.02^{A}**	**0.003 ± 0.001^{A}**	**0.24 ± 0.021^{EF}**	**0.11 ± 0.025^{AB}**	**1.2 ± 0.12^{A}**
**B3**	902541570 ± 34657093^{bcde}	142700122 ± 37193201^{b}	1142308 ± 171976^{a}	205620499 ± 65909180^{abc}	45442047 ± 7198963^{abc}	2270359 ± 566836^{ab}	28317141 ± 1652035^{abc}	773377 ± 557754^{b}	2382071 ± 133763^{ab}	1541447 ± 71150^{cde}	23907365 ± 2549614^{bcd}
%	**67 ± 6.5^{AB}**	**10.5 ± 2.12^{ABC}**	**0.08 ± 0.008^{ABC}**	**15.0 ± 4.0^{AB}**	**3.3 ± 0.33^{AB}**	**0.17 ± 0.032^{A}**	**2.1 ± 0.003^{A}**	**0.056 ± 0.038^{C}**	**0.18 ± 0.020^{ABCD}**	**0.11 ± 0.012^{AB}**	**1.8 ± 0.08^{AB}**
**B4**	733337645 ± 74541267^{abc}	133042906 ± 3270531^{b}	2558263 ± 200734^{de}	171823016 ± 2086398^{abc}	38240052 ± 1938303^{abc}	4156503 ± 217381^{bcd}	37025735 ± 2403180^{bcd}	357337 ± 130283^{ab}	1980961 ± 173030^{ab}	1229722 ± 50077^{abcd}	12066114 ± 716982^{ab}
%	**64 ± 2.9^{AB}**	**11.7 ± 0.95^{CD}**	**0.23 ± 0.005^{FG}**	**15.2 ± 1.0^{AB}**	**3.4 ± 0.36^{AB}**	**0.37 ± 0.040^{CD}**	**3.3 ± 0.40^{AB}**	**0.031 ± 0.010^{BC}**	**0.18 ± 0.025^{ABCD}**	**0.11 ± 0.011^{AB}**	**1.1 ± 0.12^{A}**
**B5**	804136125 ± 40227364^{abcde}	100982157 ± 8422559^{ab}	1978429 ± 179562^{bcd}	148529804 ± 14247976^{ab}	29554242 ± 2034746^{abc}	3573341 ± 330181^{abc}	27248066 ± 3480151^{ab}	162081 ± 17281^{ab}	1741250 ± 213838^{a}	1203493 ± 15558^{abcd}	18415325 ± 183388^{abc}
%	**71 ± 0.8^{AB}**	**8.9 ± 0.20^{ABC}**	**0.17 ± 0.005^{EF}**	**13.0 ± 0.5^{AB}**	**2.6 ± 0.02^{A}**	**0.31 ± 0.010^{BC}**	**2.4 ± 0.16^{AB}**	**0.014 ± 0.001^{AB}**	**0.15 ± 0.010^{ABC}**	**0.11 ± 0.005^{AB}**	**1.6 ± 0.11^{AB}**
**B6**	1297623398 ± 153937928^{f}	202268961 ± 4702710^{bd}	1537947 ± 91283^{abc}	212328658 ± 12374856^{bc}	52262611 ± 426677^{bcd}	3052932 ± 173244^{abc}	39025017 ± 1655587^{bcde}	218189 ± 101997^{ab}	2601717 ± 34763^{b}	1294486 ± 81170^{abcde}	21481118 ± 276979^{abc}
%	**71 ± 3.1^{AB}**	**11.1 ± 1.09^{BCD}**	**0.08 ± 0.001^{AB}**	**11.6 ± 1.6^{AB}**	**2.9 ± 0.24^{AB}**	**0.17 ± 0.003^{A}**	**2.1 ± 0.07^{A}**	**0.012 ± 0.005^{BC}**	**0.14 ± 0.009^{AB}**	**0.07 ± 0.010^{A}**	**1.2 ± 0.07^{A}**
**B7**	737501117 ± 104722371^{abc}	91056203 ± 14966619^{ab}	1482743 ± 108720^{abc}	104928027 ± 31520596^{ab}	29054400 ± 1637310^{ab}	2929451 ± 665040^{abc}	24838544 ± 4584499^{ab}	83542 ± 6726^{ab}	1983132 ± 274075^{ab}	743113 ± 198427^{a}	16790956 ± 1254191^{abc}
%	**73 ± 1.0^{B}**	**9.0 ± 0.08^{ABC}**	**0.15 ± 0.012^{DE}**	**10.3 ± 1.5^{AB}**	**2.9 ± 0.29^{AB}**	**0.29 ± 0.021^{ABC}**	**2.5 ± 0.07^{AB}**	**0.008 ± 0.001^{AB}**	**0.20 ± 0003^{BCDE}**	**0.07 ± 0.008^{A}**	**1.7 ± 0.39^{AB}**
**B8**	1068994852 ± 23244752^{ef}	122063687 ± 4286803^{b}	2127752 ± 122437^{cd}	265824225 ± 39293874^{c}	33485937 ± 3666921^{abc}	3995059 ± 132931^{bcd}	31619349 ± 737329^{abc}	177188 ± 57610^{ab}	2383771 ± 124811^{ab}	1054969 ± 40806^{abc}	18834727 ± 115241^{abc}
%	**69 ± 1.7^{AB}**	**7.9 ± 0.09^{AB}**	**0.14 ± 0.002^{CDE}**	**17.1 ± 1.7^{B}**	**2.2 ± 0.14^{A}**	**0.26 ± 0.003^{ABC}**	**2.0 ± 0.05^{A}**	**0.011 ± 0.003^{AB}**	**0.15 ± 0.001^{AB}**	**0.07 ± 0.001^{A}**	**1.2 ± 0.06^{A}**
**B9**	786239757 ± 118338835^{abcde}	227934913 ± 5599334^{d}	1701839 ± 230339^{abc}	139028903 ± 24613043^{ab}	77368869 ± 4220626^{de}	4423825 ± 259076^{cd}	50731991 ± 7429054^{de}	598940 ± 77307^{ab}	3892719 ± 45114^{c}	1894113 ± 300276^{e}	23380191 ± 6648156^{bcd}
%	**60 ± 5.3^{A}**	**17.4 ± 1.51^{E}**	**0.13 ± 0.026^{BCDE}**	**10.6 ± 2.5^{AB}**	**5.9 ± 0.69^{C}**	**0.34 ± 0.041^{BC}**	**3.9 ± 0.81^{B}**	**0.046 ± 0.009^{BC}**	**0.30 ± 0.022^{FG}**	**0.14 ± 0.032^{B}**	**1.8 ± 0.39^{AB}**
**B10**	1047140092 ± 17830892^{def}	232492424 ± 7300115^{d}	1565362 ± 262668^{abc}	129678111 ± 8817368^{ab}	95899669 ± 1443621^{ef}	8142898 ± 1287932^{e}	56436164 ± 3171211^{e}	666369 ± 43723^{ab}	3471267 ± 13590^{c}	1721907 ± 95202^{de}	24904193 ± 4639753^{cd}
%	**65 ± 1.1^{AB}**	**14.5 ± 0.46^{DE}**	**0.10 ± 0.016^{ABCD}**	**8.1 ± 0.6^{A}**	**6.0 ± 0.09^{C}**	**0.51 ± 0.080^{D}**	**3.5 ± 0.20^{AB}**	**0.042 ± 0.003^{BC}**	**0.22 ± 0.001^{DEF}**	**0.11 ± 0.006^{AB}**	**1.6 ± 0.29^{AB}**
**B11**	1019649140 ± 39507727^{cdef}	144921482 ± 20397144^{bc}	964489 ± 204627^{a}	191245724 ± 13182486^{abc}	51949320 ± 19237817^{bc}	3075796 ± 352928^{abc}	31335663 ± 7621939^{abc}	194332 ± 8127^{ab}	2053386 ± 253974^{ab}	1492655 ± 182577^{bcde}	34456252 ± 202457^{cd}
%	**69 ± 0.8^{AB}**	**9.8 ± 0.89^{ABC}**	**0.06 ± 0.011^{A}**	**12.9 ± 1.5^{AB}**	**3.5 ± 1.12^{AB}**	**0.21 ± 0.013^{AB}**	**2.1 ± 0.41^{A}**	**0.013 ± 0.001^{AB}**	**0.14 ± 0.010^{A}**	**0.10 ± 0.017^{AB}**	**2.3 ± 0.13^{B}**
**B12**	720002606 ± 60458791^{ab}	141869673 ± 13541962^{b}	3136408 ± 396934^{e}	96622336 ± 343407^{a}	114770778 ± 3969717^{f}	5596651 ± 7186^{d}	45016538 ± 4665850^{cde}	357452 ± 134274^{ab}	2391480 ± 272829^{ab}	1461570 ± 82806^{bcde}	22942230 ± 4061405^{bcd}
%	**62 ± 1.4^{AB}**	**12.3 ± 0.41^{CD}**	**0.27 ± 0.018^{G}**	**8.4 ± 0.5^{A}**	**10.0 ± 0.97^{D}**	**0.49 ± 0.030^{D}**	**3.9 ± 0.16^{B}**	**0.031 ± 0.010^{BC}**	**0.21 ± 0.011^{CDE}**	**0.13 ± 0.001^{AB}**	**2.0 ± 0.48^{AB}**
**Range (%)**	60 – 73	6.8 – 17.4	0.06 – 0.27	8.1 – 17.1	2.2 – 10	0.17 – 0.51	2.0 – 3.9	0.003 – 0.056	0.14 – 0.36	0.07 – 0.14	1.1 – 2.3
**Average (%)**	**67 ± 3.9**	**11 ± 2.9**	**0.14 ± 0.06**	**12 ± 2.9**	**4.2 ± 2.2**	**0.29 ± 0.12**	**2.7 ± 0.7**	**0.02 ± 0.02**	**0.20 ± 0.07**	**0.10 ± 0.02**	**1.56 ± 0.38**

Table S3. Peak areas obtained for each sample (average of duplicates) and considered for the statistical analysis. Peaks are numbered according to Table 1.

	***Broa* 1**	***Broa* 2**	***Broa* 3**	***Broa* 4**	***Broa* 5**	***Broa* 6**	***Broa* 7**	***Broa* 8**	***Broa* 9**	***Broa* 10**	***Broa* 11**	***Broa* 12**
**F1**	6691949	615338	1524867	2907057	9430278	697865	3007692	2254879	1869877	3858576	3342831	1006736
**F2**	3855653	760479	3129887	3127175	1382390	4510440	1352344	2784558	4916862	3907464	4613681	2871501
**F3**	1564893	477422	3770771	1089328	1697488	1321111	952693	1381521	1052209	1847419	2267586	2139840
**F4**	21688854	21250167	9601637	17453662	17648469	9818783	17879138	17560166	11664935	13661400	11023845	13449413
**F5**	2321265	1981779	2407285	2047850	2162221	1731681	3078880	2568511	3238308	3818400	2573213	3211120
**F6**	1035198	378707	715749	607644	464959	852327	825584	628717	1613649	1603575	631408	769200
**F7**	613214	405566	546782	584720	896700	501804	327758	260853	816380	842887	582574	981525
**F8**	541598	415078	291348	196372	339360	446174	140171	266720	546593	696045	202210	210884
**F9**	126038	20869	39151	46182	19503	84103	17774	37977	104130	182536	39122	61878
**F10**	5038337	3390059	5938643	3103550	2984852	5378781	4788557	3819086	6366782	5244858	4698748	3720697
**F11**	286728	187491	325055	299002	231218	375150	52497	252741	477892	465918	218651	375275
**F12**	243673923	173235728	224239502	97906758	247142336	401411249	205310585	193980303	188920874	175250853	140367303	112696099
**F13**	307535937	221549424	431424479	335784907	335995242	597568015	323162386	544448792	275385258	523653196	629111351	323870967
**F14**	4012814	3390190	3812794	5664162	2971315	4583546	4639505	6035995	6520826	9029608	3197879	6297233
**F15**	449543	305656	689062	954860	522983	559080	285260	636521	779624	952141	656882	934735
**F16**	40838453	35885319	42990320	62085584	45772044	53724887	28614781	59575993	57927654	60992699	54187945	46297816
**F17**	867065	412820	2097674	5152339	487365	745117	310839	6142932	6890261	2295240	529062	6708900
**F18**	30080154	26218804	47092492	32673318	33740731	41690627	21432196	48563747	33432526	20351278	33398063	32980737
**F19**	6387321	3504340	8036589	6809433	4394092	12544810	4362250	5490856	14379319	16203874	11434070	7005084
**F20**	40072221	50709157	40723631	44116974	42190514	61205631	73769044	77574347	55148421	70595715	39777299	65789076
**F21**	305653	393783	240636	453018	253483	241365	320697	357474	936899	1130620	394057	616611
**F22**	102038	246465	83552	1201833	652265	38535	360365	1125856	46181	208905	32905	615788
**F23**	3042589	2616761	4401703	5337709	3913548	4055291	2620028	3950549	9721303	10880388	4643749	7244604
**F24**	1479001	1330751	2246068	3213051	1727106	1733592	1068891	2317975	5272061	4816347	2265095	3603605
**F25**	11744406	364180	13408359	834582	348034	15716957	453993	559970	11943832	16644897	17052679	595660
**F26**	571613	461504	698154	1694769	609352	180361	461779	1314331	1947285	2192950	580382	1271681
**F27**	1103478	4637031	1704494	3468107	1072908	2936404	2247103	5270820	5241802	8116638	792745	599978
**F28**	279200	426296	281036	915023	329032	355566	156699	369893	469223	874177	370897	588379
**F29**	3062804	1308846	5825253	11445330	5174130	6948361	3295742	6650606	6878153	7173904	3894500	7121649
**F30**	417748	473592	261994	520235	211931	382397	449441	630675	530164	829115	246224	611802
**F31***	1582645	2730735	1646021	3365522	4945510	2295342	3356299	5253821	3730074	3660913	1482510	12986965
**F32**	9124542	5842499	18043675	34269351	19224900	25244578	13277867	33263785	30865001	32155446	22712000	16277351
**F33**	951176	624451	1382940	2906089	1020722	1807511	557674	1467504	2077353	2576416	1327184	1903610
**F34**	3282095	2289354	2054910	2823143	3805170	1721847	3549608	4082695	2201446	2851387	2559006	2472457
**F35**	197494	137709	246269	455023	147310	271196	221291	264933	242785	365691	120823	209588
**F36**	8674641	3969466	20618789	37823994	10226671	33942922	10793716	27848757	32083820	37208625	18320671	31904173
**Fo1**	1416333	917722	2188718	2625361	1836114	2916887	1769231	2083380	2617219	2819450	2349961	1412684
**Fo2**	21589399	2311547	34509222	6097889	3737198	42364060	4595524	4912079	42733413	44547955	13573723	14431175
**Fo3**	313733	207928	359807	219479	171318	267612	136672	208136	288518	301337	182264	165539
**Fo4**	223625	251073	776411	668022	300607	154510	362123	480842	4168335	4547388	815185	2415477
**Fo5**	27283680	17061444	40048767	51991492	34225043	65947012	33205283	42302007	61425654	65750920	51122357	27749381
**Fo6**	3565710	2717831	5443714	13243918	4980244	6726264	4017751	5980801	7070348	9705259	4341841	6068441
**Fo7**	1471081	746602	1774065	1784995	1235977	2402724	1166578	1550675	2590886	2864570	1977732	2254429
**Fo8**	3480035	1601196	3851382	2448360	2863359	6396902	3750601	2339302	5316795	7382578	4528913	2415935
**Fo9**	281958	156610	293402	288714	339805	271929	150199	181153	365960	183629	208990	280036
**Fo10**	2439856	1008121	2062364	1435480	1255945	3635684	1640213	1736757	2534154	3592115	2452807	1774605
**Fo11**	910587	316923	505991	254669	240466	671116	584748	119616	286959	1024379	715242	404184
**Fo12**	188622	47503	297550	171356	88889	414100	114481	119387	168235	362105	152851	77328
**Fo13**	586504	442896	357194	405198	446784	657112	603530	712418	767863	1035825	531556	804472
**Fo14**	717734	482684	1011281	1288692	593262	1264964	372989	524594	1513285	1683617	876186	1354553
**Fo15**	5393372	1463893	5250051	5421374	6488077	3183829	1359268	6888726	8928801	9793314	6347362	7284597
**Fo16**	2495148	1232673	3438660	3159910	3008151	4813505	1042741	2866037	5278547	5969719	3129652	3685929
**Fo17**	2437671	636654	1245638	2166751	1550380	3319877	1100931	2022183	4519971	3664409	2024502	2536806
**Fo18**	2258487	1350660	1878907	2585103	2598141	3113998	2271811	3227531	3643853	3997732	2921421	4413501
**Fo19**	15553948	7443467	15486010	9468744	10658071	21800415	10022706	11109564	26558986	26136459	20147214	10740090
**Fo20**	1022822	1830177	1299325	6088783	4785472	1704967	4596384	7249791	3251885	3968800	210305	9607426

**Fo21**	1276671	274500	2319515	3265982	1592619	2928300	1009895	2002399	2912255	2955028	1441297	2507664
**Fo22**	879132	391425	729777	681532	588925	1204385	630808	731231	1321939	1157641	1118531	565049
**Fo23**	1527187	683573	1201631	1001792	846184	1752950	776295	978398	2531772	1927246	1613543	976987
**Fo24**	3244663	2081387	2130452	2433202	2343718	3457588	2887274	3795027	4700976	5556173	3457370	5908237
**Fo25**	11368478	5964188	6398430	7988065	8443830	9285562	8278210	11775168	16072152	6399859	8397390	19774899
**Fo26**	11776818	3954020	7841859	5858050	5763584	11612716	4609962	6166489	16366158	15164925	10283294	12260257
**Pn1**	128721	164838	132077	118514	115605	142426	211042	141521	138760	115073	104861	99362
**Pn2**	854156	770248	891949	2299826	1751247	1306766	1223631	1830585	1421491	1263005	815491	2901688
**Pn3**	239687	143165	118282	139923	111577	88755	48071	155647	141588	187285	44138	135359
**Po1**	39416	21631	65223	65247	27185	92053	30893	45243	76576	100794	45481	38596
**Po2**	28105	21174	26837	44089	39469	32495	23171	8777	58917	50568	33806	42012
**Po3**	6576263	482803	7836481	709701	1018669	10989002	943673	1167002	13469699	11253499	10162696	2503960
**Po4**	863864	713807	1377747	2505068	1519070	1843557	1369072	1783058	2355182	2048686	1149389	1929465
**Po5**	987323	1089567	653389	886066	1009525	890442	1068723	1123177	847614	853004	747275	840535
**Po6**	739294	496635	461982	796668	891235	1207374	853089	1004493	1033968	1016578	956892	628915
**Po7**	107105240	109561012	171474298	115185328	115644962	163141630	79813166	192598823	91506723	83230534	125669543	57134529
**Po8**	8331390	14438103	21355513	46454551	24388393	31001539	17848051	63810414	26214143	27939120	50536109	27221265
**Po9**	26697	5809	84414	59774	34441	54675	12704	27734	62404	54605	15556	10246
**Pz1**	1593679	837242	1418950	1625846	860505	1668279	957239	1055195	2705312	2838027	996329	3019652
**Pz2***	11366618	6623650	10100276	13871874	9133466	12323112	8176939	12291280	22040199	28520900	9606416	45230718
**Pz3**	30531761	7618190	24334797	12123377	10583455	26950791	10864691	10338605	35309745	44011053	31747791	36087651
**Pz4**	1084374	1416652	1339397	1130510	1215550	1123698	2386891	1370981	2850949	3171803	1104668	7593796
**Pz5**	1110181	1481207	1431379	2380468	1799076	1985463	1511212	1668634	3512562	4218684	1700557	6168152
**Pz6**	4867019	2559376	4047867	4621055	4444498	4712331	3156703	4061757	6938815	8746235	4399120	10522425
**Pz7**	1325194	348275	913191	436317	235384	861382	516394	455157	1446934	1646952	939177	1894102
**Pz8**	620205	236437	256309	413386	111582	424409	194709	598426	553190	827785	186801	602217
**Pz9**	1194268	231396	852879	431641	346272	1155999	778895	683574	726667	246223	382162	1967528
**Pz10**	199414	75250	118028	203713	237314	169824	107191	141060	251290	353318	177017	451967
**Pz11**	391966	228872	369168	626039	464914	528529	304595	548245	633890	766596	468103	807122
**Pz12**	314141	102980	259807	375830	122228	358797	98943	273025	399318	552095	241181	425452
**Pd1**	257752	110243	251056	310865	235471	374522	150685	268402	668170	710614	284372	571485
**Pd2**	22664	8855	4387	5361	9068	3789	5140	3410	7617	8456	6430	24087
**Pd3**	336126	108250	226514	246390	255700	355284	258639	276468	414519	474652	477590	245480
**Pd4**	94098	73629	107609	126738	94380	128577	82481	136483	223971	211331	77727	271447
**Pd5**	38838	34892	36190	49097	23323	28660	15629	22958	24706	34289	23656	25099
**Pd6**	41370	18579	218063	118391	95460	107907	232797	205511	311505	163167	173208	321905
**Pd7**	120531	474230	424975	872845	655666	691329	693501	900879	1250385	4973064	794265	1848534
**Pd8**	860844	819664	967771	2323907	2107323	1281307	1422393	2081962	1418476	1434034	1180093	2156897
**Pd9**	127585	37237	33794	102910	96952	81559	68186	98988	104479	133293	58458	131719
**Py1**	105715	79069	66900	39302	19828	78637	62546	43250	81720	93742	42327	107957
**Py2**	715664	506628	785394	898007	589260	760489	413685	750007	1302663	1533553	685704	1246135
**Py3**	573938	363224	687486	481540	309593	639073	462350	491480	1396012	1187878	410619	970965
**Py4**	2202592	951949	2167007	3642265	698738	2753303	958906	1225934	3359658	3332886	1510001	2901475
**Py5**	241791	43798	275423	490598	236390	579406	33728	315608	596795	681427	299826	187009
**Py6**	21342173	9004823	17160728	20235766	16263386	26353008	13456932	16950393	32633721	35698940	22526622	25047325
**Py7**	289382	402083	127891	68465	67201	411181	116514	130409	186503	481574	93345	142034
**Py8**	348245	257967	1540564	537328	329821	440653	477825	573086	2480093	3421058	296613	1247075
**Py9**	397774	548147	561906	1285431	496122	804005	487235	915915	1040690	1267756	294266	1207833
**Py10**	3736442	6238638	4111672	8043309	7456520	5135785	7685556	9398938	6091910	6559038	4297102	9631639
**Py11**	477967	321395	463463	891510	582781	702182	590217	641636	1151719	1635629	679147	1549511
**Py12**	90463	13094	43289	28958	18707	64934	15544	29492	76551	68983	38108	120864
**Py13**	241070	124600	325418	383259	179722	302364	77510	153205	333960	473703	161985	656718
**Ox1**	52214	13395	38277	8894	4430	35407	24683	9388	67321	70531	4430	28355
**Ox2**	72555	1085	551104	14116	7058	7058	7058	7058	299659	289879	19229	33775
**Ox3**	120395	7962	183996	334327	150593	175724	51801	160743	231961	305959	170673	295323
**Tz1**	652472	198748	554124	61430	44492	446190	322918	43604	500951	502178	14762	187477
**Tz2**	898987	954719	867782	1356396	1117765	807859	1080597	1473119	1404647	1602954	780704	1520775
**Tz3**	2473991	825779	960165	563136	578994	1347668	579617	867049	1987122	1366136	1257920	683228
**Tp1**	23851	12412	24036	19935	12314	9617	34909	13451	33196	36899	19646	28521
**Tp2**	274934	291071	360986	151372	413347	197151	131751	95375	201387	153420	583758	178681
**Tp3**	701088	430162	815657	828866	518663	803099	384527	680956	1235079	1148656	656055	942946

**Tp4**	102411	87452	160409	105888	77205	108464	83873	108783	191958	177546	104739	177125
**Tp5**	80581	48045	72588	76253	36419	73881	45258	48791	102897	110820	81453	73353
**Tp6**	127348	47125	107771	47409	145545	102275	62797	107615	129597	94568	47004	60945
**S1**	5183873	3682260	5638402	5201407	7695280	6003347	6389799	9573231	5550541	5467429	6541864	6459281
**S2**	706027	1563140	1854872	1463810	2508621	748310	2458145	2707726	1195649	2555254	1543694	971464
**S3**	7807314	3373056	14252565	4245342	6942347	10015802	5212575	4735382	11739271	10865322	20073686	10846713
**S4**	98446	97302	100179	71783	58688	112405	100532	87601	133405	123900	88229	138477
**S5**	30161	13520	63274	13355	13628	13142	2591	2591	13499	14101	22650	12333
**S6**	40737	146590	20376	110645	115345	11715	78222	52739	13040	17339	8077	76373
**S7**	124612	140916	35734	342249	233037	1677600	1379107	494292	1555969	1604271	1401192	689287
**S8**	1204280	518964	1941963	617525	848380	2898799	1169987	1181168	3178819	4256579	4776862	3748305

*: Peak areas of B12 are significantly different ($p < 0.05$).

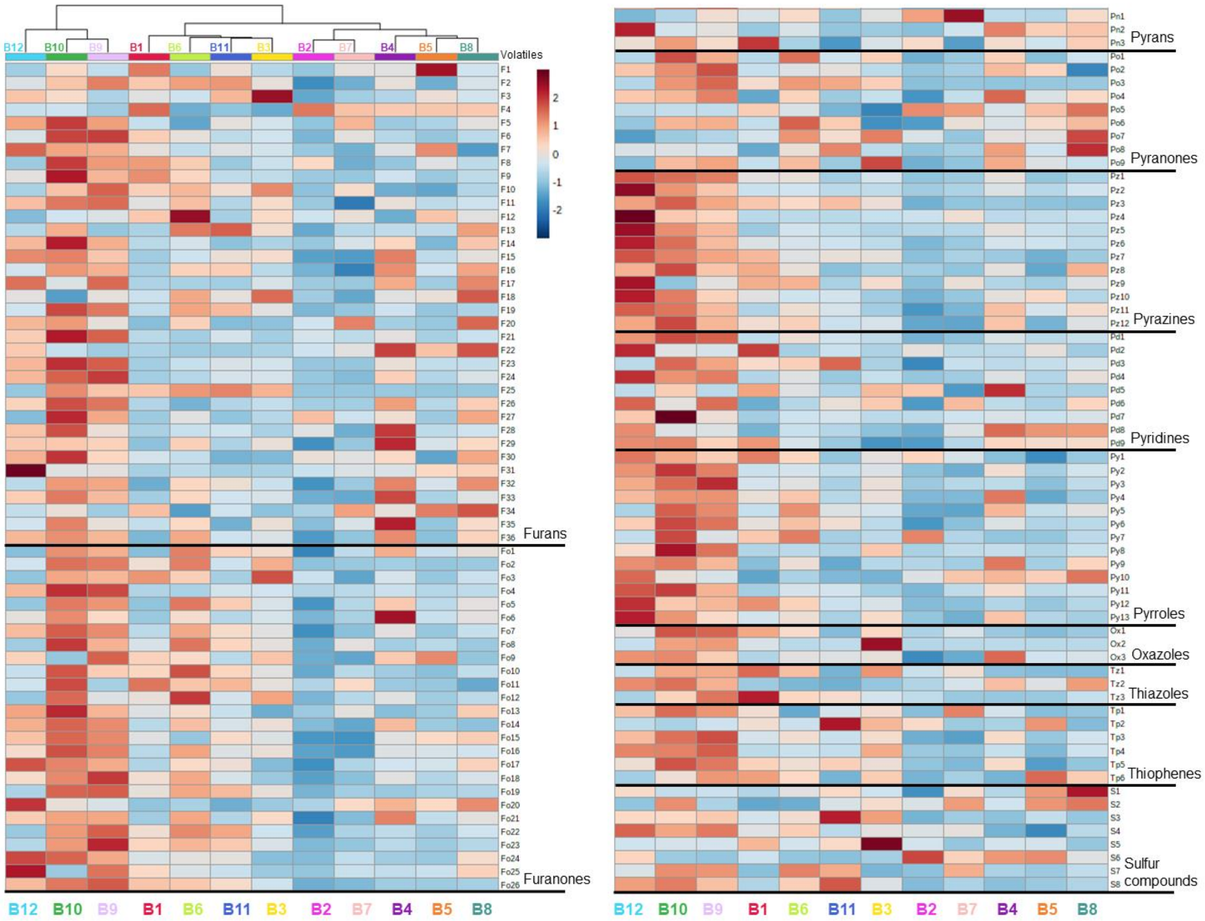

Figure S4. Heatmap and hierarchical cluster analysis representation of the 128 volatiles identified in *broas*. The content of each metabolite is illustrated through a chromatic scale (from deep blue, minimum, to deep red, maximum).

Tables S4 and S5. Spearman correlation coefficients among the 128 studied *broas'* volatile compounds.

Cf. Excel File "**Tables S4 and S5**".
